# The BAFF Receptor Transduces Survival Signals by Co-opting the B Cell Receptor Signaling Pathway

**DOI:** 10.1016/j.immuni.2012.11.015

**Published:** 2013-03-21

**Authors:** Edina Schweighoffer, Lesley Vanes, Josquin Nys, Doreen Cantrell, Scott McCleary, Nicholas Smithers, Victor L.J. Tybulewicz

**Affiliations:** 1 Division of Immune Cell Biology, MRC National Institute for Medical Research, London NW7 1AA, UK; 2 University of Dundee, Dundee DD1 4HN, UK; 3 Immuno-Inflammation Therapy Area Unit, GlaxoSmithKline, Stevenage SG1 2NY, UK

## Abstract

Follicular B cell survival requires signaling from BAFFR, a receptor for BAFF and the B cell antigen receptor (BCR). This “tonic” BCR survival signal is distinct from that induced by antigen binding and may be ligand-independent. We show that inducible inactivation of the Syk tyrosine kinase, a key signal transducer from the BCR following antigen binding, resulted in the death of most follicular B cells because Syk-deficient cells were unable to survive in response to BAFF. Genetic rescue studies demonstrated that Syk transduces BAFFR survival signals via ERK and PI3 kinase. Surprisingly, BAFFR signaling directly induced phosphorylation of both Syk and the BCR-associated Igα signaling subunit, and this Syk phosphorylation required the BCR. We conclude that the BCR and Igα may be required for B cell survival because they function as adaptor proteins in a BAFFR signaling pathway leading to activation of Syk, demonstrating previously unrecognized crosstalk between the two receptors.

## Graphical abstract

**Graphical Abstract:**

**Figure:**
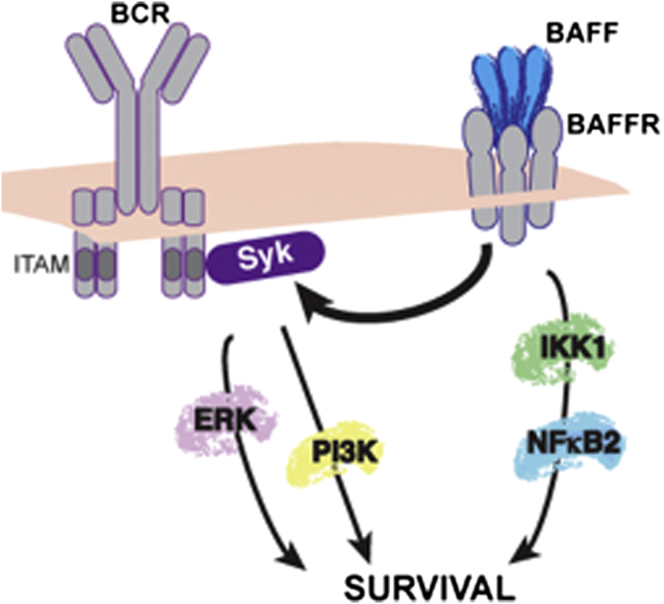

**Highlights:**

► Inducible loss of the Syk tyrosine kinase results in death of follicular B cells ► Syk transduces survival signals from BAFFR to the ERK and PI3 kinase-PDK1 pathways ► BAFFR signaling results in phosphorylation of Igα and Syk ► BAFFR transduces signals via the BCR to activation of Syk

## Introduction

B lymphocytes play a critical role in the adaptive immune response, in part by producing high affinity antibodies to pathogens. There are at least three main lineages of mature B cells. Recirculating follicular B cells reside in the follicles of secondary lymphoid organs and traffic between them through the blood and lymphatic circulations; marginal zone (MZ) B cells are located in the periphery of the splenic white pulp and are largely nonrecirculating; B1 cells are found predominantly in the peritoneal and pleural cavities. The total number of mature naive (unactivated) B cells remains largely constant despite continuous production of new B cells in the bone marrow as well as recruitment of naive B cells into antigen-activated compartments, such as germinal center cells, plasma cells, and memory B cells. This homeostasis of mature B lymphocytes is known to depend on at least two receptors: BAFFR (TNFRSF13C) and the B cell antigen receptor (BCR).

Mice deficient in BAFFR or its ligand BAFF (TNFSF13B) have substantially reduced numbers of follicular and MZ B cells, but unaltered numbers of B1 cells (Gross et al., 2001; Mackay et al., 2010; Miller and Hayes, 1991; Sasaki et al., 2004; Schiemann et al., 2001; Schneider et al., 2001; Shulga-Morskaya et al., 2004; Thompson et al., 2001). Furthermore, treatment of mice with reagents that block binding of BAFF to BAFFR leads to loss of most follicular cells, whereas transgenic elevation of BAFF expression leads to increased numbers of B cells (Gross et al., 2000, 2001; Mackay et al., 1999). Thus BAFF regulates B cell survival, and the amount of BAFF determines the size of the B cell compartment. Studies have shown that BAFFR signals in part through the TRAF2 and TRAF3 E3 ligases, leading to activation of the MAP 3-kinase NIK and IκB kinase 1 (IKK1). This promotes the proteolytic processing of NF-κB2 (p100) into p52, an NF-κB family transcription factor that translocates into the nucleus and regulates gene expression (Rickert et al., 2011).

On mature B cells, the BCR is found in the form of surface-bound immunoglobulin M (IgM) and IgD. These proteins are both associated with the nonpolymorphic Igα and Igβ (CD79a and CD79b) transmembrane proteins, which are required for BCR signal transduction (Kurosaki, 1999). Inducible loss of the BCR or Igα results in the rapid death of all subsets of mature B cells (Kraus et al., 2004; Lam et al., 1997). Furthermore, B cells are also lost following deletion of a portion of the cytoplasmic domain of Igα containing an immunoreceptor tyrosine-based activation motif (ITAM), which is critical for signaling from the BCR (Kraus et al., 2004). These results suggest that the BCR delivers a signal required for the survival of B cells. Such a signal could be generated either following low-affinity interactions of the BCR with self-antigens, or by continuous low-level “tonic” BCR signaling in the absence of ligand engagement. Survival of BCR-deficient B cells can be rescued by ectopic activation of phosphatidylinositide-3 (PI3) kinase and this survival signal may be mediated in part by Akt, which phosphorylates and inactivates the FOXO1 transcription factor, a regulator of proapoptotic genes. Taken together, these results suggest that the BCR transduces a B cell survival signal via PI3 kinase, Akt, and FOXO1 (Srinivasan et al., 2009). However, because BAFFR can directly lead to PI3 kinase and Akt activation (Otipoby et al., 2008; Patke et al., 2006; Woodland et al., 2008), it remains unclear why B cell survival requires signals from both the BCR and BAFFR.

Whereas the BCR delivers a survival signal in resting mature B cells, antigen binding to the receptor promotes B cell activation, proliferation, and differentiation. Thus signaling from the BCR can lead to two quite different outcomes. However the mechanism underlying these differences is unknown. Binding of antigen to the BCR leads to rapid phosphorylation of two tyrosines within the ITAMs of Igα and Igβ, probably mediated by Src-family kinases (Kurosaki, 1999). Subsequently, the tandem phosphotyrosines on the Igα and Igβ ITAMs serve as binding sites for the tandem SH2 domains of the Syk tyrosine kinase, leading to autophosphorylation and activation of the kinase (Mócsai et al., 2010). Studies in the chicken DT40 leukemic B cell line have shown that loss of Syk blocks BCR-induced calcium flux, suggesting that Syk is critical for the antigen-induced activation signal from the BCR (Takata et al., 1994). Deletion of the *Syk* gene in mice results in a partial block in B cell development at the pre-BCR checkpoint and complete arrest at the BCR checkpoint. Consequently, no mature B cells develop at all, consistent with a key role for Syk in transducing pre-BCR and BCR signals required for developmental progression (Cheng et al., 1995; Turner et al., 1997; Turner et al., 1995). In view of these findings, it is possible that Syk may also be important for the tonic BCR survival signal. Here we investigated this possibility by studying the effect of inducible deletion of *Syk* on B cell survival.

We found that deletion of *Syk* led to loss of follicular and MZ B cells, which correlated with the inability of Syk-deficient B cells to survive in response to BAFF. By using biochemical and genetic rescue approaches, we demonstrated that Syk transduces key BAFFR survival signals via the ERK and PI3 kinase pathways. Surprisingly, we discovered that BAFF stimulated rapid phosphorylation of Igα and Syk and that BAFF-induced phosphorylation of Syk required the BCR. Thus we conclude that the BAFFR and BCR signaling pathways are closely connected and that rather than delivering an independent tonic survival signal, the BCR and its associated Igα subunit may serve as adaptor proteins in a BAFFR signaling pathways required for B cell survival.

## Results

### Loss of Syk Results in Loss of Most Mature B Cells

**Figure 1:**
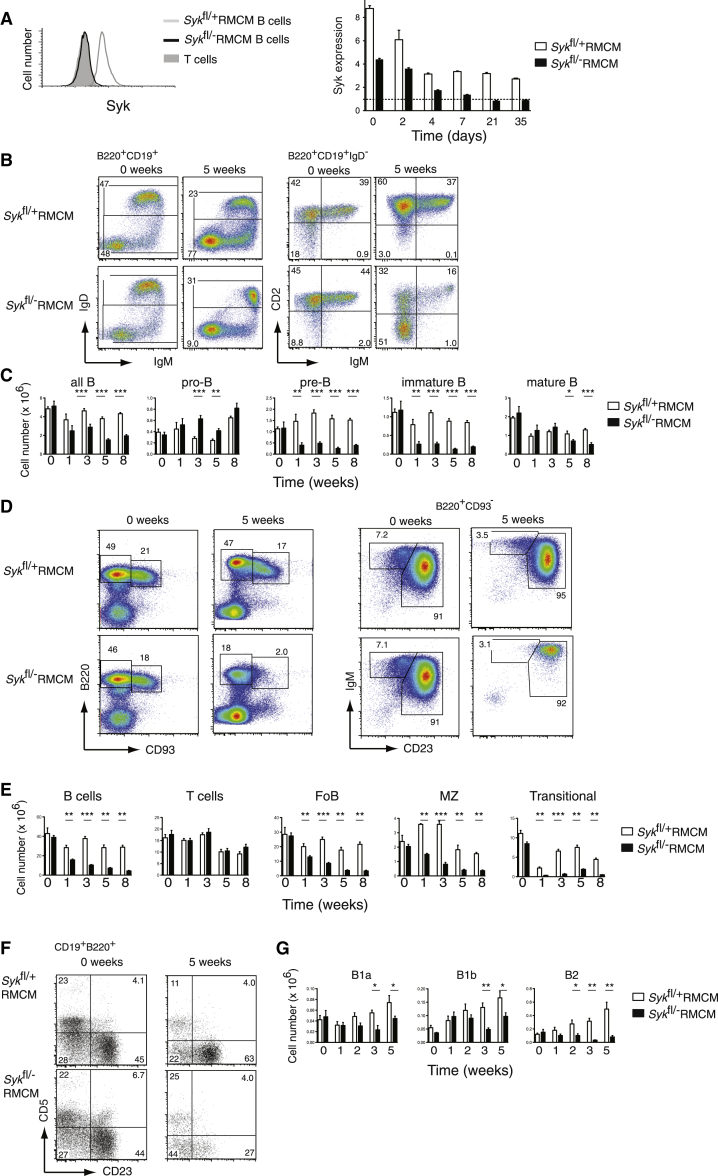
Deletion of Syk Leads to the Loss of Most, but Not All, B Lineage Cells Control (*Syk*^fl/+^;*Rosa26*^MerCreMer/+^, *Syk*^fl/+^RMCM) or conditional Syk-deficient mice (*Syk*^fl/−^;*Rosa26*^MerCreMer/+^, *Syk*^fl/−^RMCM) were treated with tamoxifen. (A) The left panel shows a histogram of the amount of intracellular Syk protein in B or T cells from indicated mice 3 weeks after start of tamoxifen injections. The right panel shows the mean (±SEM) fluorescence intensities of anti-Syk antibody, normalized to signal in T cells (dashed line) in mice of indicated genotype as a function of time following tamoxifen injection. Note that the control mice start with two functional *Syk* alleles, which is reduced to one following tamoxifen treatment, and conditional Syk-deficient mice start with one allele, which is reduced to no functional alleles following tamoxifen treatment. Hence the conditional mice start with about half the amount of Syk protein compared to control mice. (B) Flow cytometric analysis of bone marrow B lineage cells (B220^+^CD19^+^ or B220^+^CD19^+^IgD^-^) before and 5 weeks after tamoxifen treatment from indicated mice. Numbers indicate percentages of cells in the marked gates. (C) Graph of mean (±SEM) number of all B lineage cells (B220^+^CD19^+^), pro-B (B220^+^CD19^+^IgD^−^IgM^−^CD2^−^), pre-B (B220^+^CD19^+^IgD^−^IgM^−^CD2^+^), immature B (B220^+^CD19^+^IgD^−^IgM^+^CD2^+^), and mature B (B220^+^CD19^+^IgD^+^) cells in the bone marrow of indicated mice as a function of time following tamoxifen injection, determined using gates shown in (B). (D) Flow cytometric analysis of splenic B lineage cells before and 5 weeks after tamoxifen treatment from indicated mice. Left-hand panels identify transitional (B220^+^CD93^+^) and mature (B220^+^CD93^−^) B cells; right-hand panels show separation of mature B cells into follicular B cells (CD23^+^IgM^+^) and marginal zone B cells (CD23^−^IgM^+^). Numbers indicate percentages of cells in the marked gates. (E) Graph of mean (±SEM) number of B cells (B220^+^), T cells (CD4^+^ or CD8^+^), and follicular (FoB, B220^+^CD93^−^CD23^+^IgM^+^), marginal zone (MZ, B220^+^CD93^−^CD23^−^IgM^+^), and transitional (B220^+^CD93^+^) B cells in the spleen of indicated mice as a function of time following tamoxifen injection, determined using gates shown in (D). (F) Flow cytometric analysis of B cells (B220^+^CD19^+^) from the peritoneal cavity before and 5 weeks after tamoxifen treatment from indicated mice. Numbers indicate percentages of cells in the marked gates. (G) Graph of mean (±SEM) number of B1a (B220^+^CD19^+^CD5^+^CD23^−^), B1b (B220^+^CD19^+^CD5^−^CD23^−^), and B2 (B220^+^CD19^+^CD5^−^CD23^+^) cells in the peritoneal cavity of indicated mice as a function of time following tamoxifen injection, determined using gates shown in (F). See also Figure S1.

To investigate the potential role of Syk in transducing the BCR tonic survival signal, we utilized a mouse strain in which *Syk* could be inducibly deleted. This consisted of a conditional allele of *Syk* in which exon 11 is flanked by loxP sites (*Syk*^fl^) and an allele of ROSA26 (*Rosa26*^MerCreMer^) expressing a tamoxifen-inducible MerCreMer fusion protein consisting of the Cre recombinase fused to two mouse estrogen receptor hormone-binding domains (Zhang et al., 1996). We generated two mouse strains: control (*Syk*^fl/+^*Rosa26*^MerCreMer/+^, *Syk*^fl/+^RMCM) or conditional Syk-deficient mice (*Syk*^fl/−^*Rosa26*^MerCreMer/+^, *Syk*^fl/−^RMCM). Both strains of mice expressed the Cre recombinase and had one loxP-flanked allele of *Syk* but differed in the second *Syk* allele, with the control mice having a wild-type allele (*Syk*^+^) and the Syk-deficient mice having a nonfunctional deleted allele (*Syk*^−^). This allowed us to control for the activity of the recombinase and any potential effect of deleting and recombining genomic DNA. Treatment of conditional Syk-deficient mice with tamoxifen resulted in the loss of Syk in virtually all B cells, with the amount of Syk falling below detection by 10 days following start of tamoxifen treatment (Figure 1A). In contrast, treatment of control mice with tamoxifen resulted in loss of one allele of *Syk* and thus protein amounts fell by about half.

Analysis of B lineage cells in these mice showed that loss of Syk caused no change in the number of bone marrow pro-B cells but resulted in a rapid loss of bone marrow pre-B and immature B cells and splenic transitional B cells (Figures 1B–1E), resembling the phenotype of mice with a constitutive deficiency of Syk, which show a developmental block at the pre-BCR checkpoint and a block in BCR-driven positive selection of immature B cells into the mature B cell compartment (Cheng et al., 1995; Turner et al., 1995, 1997). Furthermore, we saw a loss of the majority of marginal zone B cells and most (∼80%) follicular B cells (Figures 1D and 1E). Most recirculating follicular B cells were also lost from blood and lymph nodes (data not shown); however, around 50% of control numbers of mature recirculating B cells remained in the bone marrow (Figures 1B and 1C). Lastly, we examined B cells in the peritoneal cavity. In agreement with the loss of follicular B cells in the spleen, we saw a loss of the equivalent B2 cells. However B1 cells (both B1a and B1b) were only partially reduced (Figures 1F and 1G). In contrast, the number of T cells was unaffected by the inducible loss of Syk (Figure 1E). Taken together, these results show that inducible loss of Syk in an adult mouse resulted in B cell developmental blocks in the bone marrow at the pre-BCR and BCR checkpoints and caused the disappearance of most mature follicular and marginal zone B cells, though not of B1 cells. It is unlikely that this loss of B cells is a consequence simply of a block in B cell development, because in the absence of input from the bone marrow, mature B cells turn over with a half-life of around 4.5 months (Hao and Rajewsky, 2001), whereas loss of Syk caused the disappearance of most mature B cells within 3 weeks.

We analyzed the characteristics of the surviving pool of Syk-deficient B cells. Similar to control mature B cells, these cells were largely nondividing, turned over at an even slower rate and persisted for at least 8 weeks from the start of tamoxifen treatment (see Figures S1A and S1B available online; Figure 1E). Phenotyping of cell surface markers showed that these surviving cells most closely resembled follicular B cells, being B220^+^CD19^+^IgM^+^IgD^+^CD23^+^CD21^+^MHCclassII^+^CD40^+^CD1d^−^CD93^−^ (Figure S1C). However, they expressed much more surface IgM and somewhat more CD19. It is unlikely that this represents the selective survival of a subset of B cells with high amounts of IgM, because this increase in IgM could be seen already 4 days after the start of tamoxifen treatment, before any decrease in B cell numbers (data not shown). Rather, the loss of Syk may cause a change in the recycling of IgM between the cell surface and the cytoplasm.

### Requirement for Syk in B Cell Survival Is Intrinsic to the B Cell Lineage

We next investigated whether the requirement for Syk in B cell survival is cell intrinsic. This was an important issue because Syk is expressed in most hematopoietic lineages and the *Rosa26*^MerCreMer^ allele is expressed ubiquitously, and thus loss of Syk could affect B cell survival indirectly through other cell types. We reconstituted the hematopoietic system of irradiated mice with a mixture of wild-type (Ly5.1+) and either control or conditional Syk-deficient bone marrow cells (Ly5.2+). Eight weeks later, once the mice were fully reconstituted, they were treated with tamoxifen and B cell numbers were followed for 8 weeks. Results showed that there was a selective loss of B cells that had lost Syk, even in the presence of wild-type B cells (Figure S1D). Once again, the small surviving population of Syk-deficient B cells most closely resembled follicular B cells, except for elevated amounts of IgM (Figure S1E). In a second approach, we bred the conditional *Syk* allele to mice expressing the tamoxifen-inducible Cre recombinase Cre-ERT2 under the control of the B lineage-specific *Cd79a* promoter (*Cd79a*^CreERT2^). As before, we found that 3 weeks after the start of tamoxifen treatment pre-B and immature B cells were lost from the bone marrow, all transitional and marginal zone B cells and around 80% of follicular B cells were lost from the spleen, whereas most B1 cells persisted in the peritoneum (Figures S1F–S1I). The surviving Syk-deficient B cells expressed the characteristically high amounts of IgM described before (Figures S1F and S1G). Taken together these results demonstrate that the requirement for Syk in B cell survival is cell autonomous and intrinsic to the B lineage itself. This loss of B cells after deletion of Syk is similar to that reported in mice in which the BCR is deleted (Kraus et al., 2004; Lam et al., 1997), consistent with the hypothesis that Syk transduces the tonic BCR signal required for cell survival.

### Syk-Deficient B Cells Are Unable to Survive in Response to BAFF

**Figure 2:**
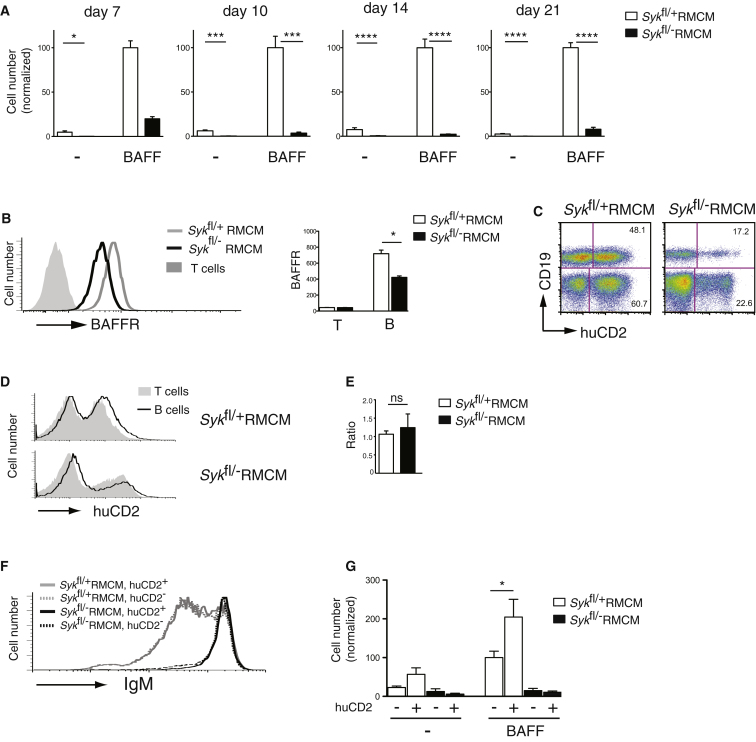
Syk-Deficient B Cells Are Unresponsive to BAFF, a Defect that Is Not Reversed by Ectopic Expression of BAFFR (A) Graph showing mean (±SEM) number of B cells of the indicated genotypes surviving after 4 days culture in the absence (-) or presence of BAFF, normalized to the number of surviving control B cells in presence of BAFF. Cells were harvested 7–21 days after tamoxifen injection, as indicated. (B) Histograms of BAFFR amounts on splenic B cells (IgM^+^) from mice of the indicated genotypes treated with tamoxifen 21 days earlier, and on T cells from *Syk*^fl/−^RMCM mice as a negative control. Graph shows mean (±SEM) BAFFR amounts on B and T cells from the indicated mice. (C and D) Irradiated mice were reconstituted with *Syk*^fl/+^RMCM or *Syk*^fl/−^RMCM bone-marrow cells, previously infected with a retrovirus expressing BAFFR and the human CD2 extracellular and transmembrane domains (huCD2), treated with tamoxifen and analyzed 6 weeks later. Flow cytometric analysis of donor splenic B and T cells from the chimeras: dot plot of CD19 and huCD2 expression (C) and histograms of huCD2 expression (D). Numbers indicate percentage of cells falling into quadrants. (E) Graph of mean (±SEM) ratio of percentage of huCD2^+^ B cells to percentage of huCD2^+^ T cells in the spleens of mice of the indicated genotypes (ns, not significantly different). Increase in this ratio would imply a survival advantage for the transduced B cells. (F) Histograms of surface IgM amounts on donor splenic B cells from chimeras described in (C) subdivided according to huCD2 expression, as a marker of retroviral infection. (G) Graph showing mean (±SEM) number of donor B cells from chimeras reconstituted with the indicated genotypes surviving after 4 days culture in the absence (-) or presence of BAFF. Cells were subdivided according to expression of huCD2. Numbers are normalized to the number of surviving control huCD2^−^ B cells in presence of BAFF. See also Figure S2.

To investigate the mechanism by which Syk contributes to B cell survival, we examined the ability of Syk-deficient B cells to survive in vitro in response to BAFF, a cytokine critically required for the survival of follicular and marginal zone B cells (Mackay et al., 2010). We found that Syk-deficient B cells were very defective in their ability to survive in response to BAFF (Figure 2A; Figure S2A). This defect was not due to the phenotype of an atypical subtype of B cells that survives in the absence of Syk, because a survival defect in response to BAFF could be seen as soon as Syk protein starts to be lost from the cells. For example, 7 days after the start of tamoxifen the number of B cells had not yet changed greatly, but most Syk had been lost and the B cells were already substantially impaired in their ability to respond to BAFF (Figure 2A). By 10 days following tamoxifen treatment, Syk-deficient B cells showed almost no BAFF-induced survival (Figure 2A), yet gene expression analysis showed only very small differences in expression between mutant and control B cells (4 differentially expressed genes out of 21,000) supporting the view that the Syk-deficient B cells were not an unusual subset of B cells (Figure S2B). In view of the importance of BAFF for B cell survival, it is thus very likely that the loss of follicular and marginal zone B cells in the absence of Syk is due to an inability of the cells to respond to BAFF. In agreement with this hypothesis, we note that B1 cells do not require BAFF for survival (Mackay et al., 2010), and this is the subset that is least dependent on Syk for its survival.

### Loss of Syk-Deficient B Cells Is Not Due to Reduced Expression of BAFFR

It has been proposed that signaling through the BCR leads to an increase in expression of BAFFR (Smith and Cancro, 2003). Thus one possible reason for the inability of Syk-deficient B cells to survive in response to BAFF is that Syk normally transduces BCR signals required to maintain BAFFR expression. However, flow cytometric analysis showed only a small decrease in BAFFR in Syk-deficient B cells to ∼60% of control amounts (Figure 2B). Nevertheless, to evaluate whether this decrease affected BAFF-induced survival, we established a retroviral-based complementation procedure to ectopically increase BAFFR expression. Initially, we tested the procedure by using a retroviral vector expressing Syk and GFP (as a marker of infection) to infect bone marrow cells from control (*Syk*^fl/+^RMCM) or conditional Syk-deficient mice (*Syk*^fl/-^RMCM), which were used to reconstitute irradiated mice. Once reconstitution was complete (around 8 weeks posttransplant), the chimeric mice were treated with tamoxifen and analyzed 6 weeks later. B cells from the conditional Syk-deficient mice infected with the retrovirus (GFP^+^) expressed about 40% the amount of Syk found in control B cells (Figure S2C). This amount of expression was sufficient to allow enhanced survival compared to uninfected (GFP^−^) Syk-deficient B cells, as seen by a higher ratio of GFP^+^ to GFP^−^ conditional Syk-deficient B cells compared to the ratio of GFP^+^ to GFP^−^ T cells in the same animal (Figures S2D–S2F). Ectopic expression of Syk in Syk-deficient B cells also corrected the surface IgM amounts, reducing them to levels similar to those seen in control B cells, and partially rescued the defect in BAFF-induced survival (Figures S2G and S2H). In contrast, retroviral-driven Syk expression in control B cells did not provide a selective survival advantage, presumably because the amount of Syk expressed from the endogenous *Syk* locus was not limiting for survival and did not change amounts of IgM or affect BAFF-induced cell survival (Figures S2D–S2H).

We also used the same retroviral transduction system to ectopically express a kinase dead form of Syk in both control and conditional Syk-deficient B cells. Following deletion of the endogenous *Syk* gene, this mutant Syk protein was unable to confer a selective in vivo survival advantage to B cells that had lost expression of endogenous Syk, did not normalize surface IgM amounts and did not rescue the BAFF-induced survival defect (Figures S2C–S2H). Taken together these results show that retroviral gene transduction of Syk into bone marrow cells from conditional Syk-deficient mice rescues B cell survival and thus this system can be used to test the ability of other genes to rescue survival of Syk-deficient B cells. We also conclude that the kinase activity of Syk is essential for its function in mediating B cell survival.

In order to determine whether the reduced expression of BAFFR on Syk-deficient B cells was the cause of their failure to survive in response to BAFF, we used a retroviral vector expressing BAFFR and a truncated form of human CD2 (as a marker of infection) to increase receptor amounts on both control and conditional Syk-deficient B cells. We found that increased expression of BAFFR did not promote increased survival of Syk-deficient B cells in vivo, had no effect on the increased amounts of surface IgM and did not rescue the defect in BAFF-induced survival in vitro (Figures 2C–2G). In contrast, increased expression of BAFFR on control B cells increased survival in response to BAFF in vitro, demonstrating that the increased amounts of the receptor were functional. Thus, the reduced expression of BAFFR on Syk-deficient B cells is unlikely to account for their failure to survive.

### Reduced Survival of Syk-Deficient B Cells Is Not Caused by Defects in the NF-κB2 p100 Pathway

**Figure 3:**
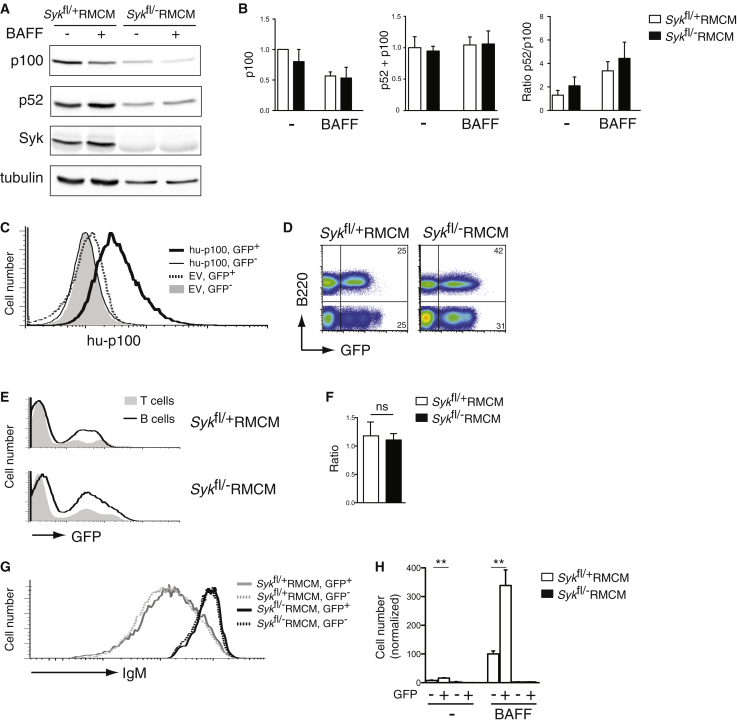
Ectopic Expression of p100 Does Not Rescue Defect in BAFF-Induced Survival of Syk-Deficient B Cells (A) Immunoblot of total cell extracts from splenic B cells of the indicated genotypes that had been cultured for 16h in the absence (-) or presence (+) of BAFF, probed with antibodies to NFκB2 (to detect p100 and p52), Syk and tubulin. (B) Graphs of mean (±SEM) amounts of p100, p52+p100, and ratio of p52/p100 from splenic B cells as described in (A). p100 and p52 amounts were normalized to tubulin and then p100, p52+p100, and the ratio of p52/p100 were normalized to control B cells cultured without BAFF. (C) Histogram shows amounts of human p100 (hu-p100) in B cells from radiation chimeras reconstituted with bone marrow of *Syk*^fl/+^RMCM mice infected with empty vector (EV) or retrovirus expressing hu-p100; both vectors express GFP. Cells were subdivided according to GFP expression. (D and E) Flow cytometric analysis of donor splenic B and T cells from radiation chimeras reconstituted with bone marrow of *Syk*^fl/+^RMCM or *Syk*^fl/−^RMCM mice infected with retrovirus expressing hu-p100 and GFP, then treated with tamoxifen to induce deletion of Syk. Numbers indicate percentage of cells falling into quadrants. (F) Graph of mean (±SEM) ratio of the percentage of donor B cells that were GFP^+^ to the percentage of donor T cells that were GFP^+^ in the spleens of chimeric mice reconstituted with bone marrow of the indicated genotypes (ns, not significantly different). (G) Histograms of surface IgM amounts on donor splenic B cells from chimeras described in (B) subdivided according to GFP expression, as a marker of retroviral infection. (H) Graph showing mean (±SEM) number of donor B cells from chimeras reconstituted with the indicated genotypes surviving after 4 days culture in the absence (-) or presence of BAFF. Cells were subdivided according to expression of GFP. Numbers are normalized to the number of surviving control GFP^−^ B cells in presence of BAFF. See also Figure S3 and Table S1.

A critical signaling pathway from BAFFR required for B cell survival involves phosphorylation of NF-κB2 p100 by IKK1, its processing into p52 and subsequent nuclear translocation of the p52 transcription factor (Rickert et al., 2011). It has been proposed that the BCR tonic signal through Syk is required for the synthesis of p100, which is then used in an IKK1 signaling pathway emanating from BAFFR (Stadanlick et al., 2008). Such a pathway could potentially explain the dual requirement for both the BCR and BAFFR for B cell survival. We examined this possibility by measuring amounts of both p100 and p52 in control and mutant B cells before and after BAFF stimulation. For this and subsequent biochemical studies, we used B cells from mice treated with tamoxifen 10 days earlier because by this time point there was no detectable Syk expression, most of the cell death had not yet occurred and in their transcriptome the cells still closely resembled control B cells (Figure S2B). We found that unstimulated Syk-deficient B cells had slightly lower p100 amounts than control B cells, but unaltered total amounts of p52+p100 protein (Figures 3A and 3B) or NF-κB2 messenger RNA (mRNA) (data not shown). However, in response to BAFF the amounts of p100 dropped and the ratio of p52/p100 rose in both control and mutant B cells, suggesting that the p100 pathway was functioning in the absence of Syk (Figures 3A and 3B). Nevertheless, to test this hypothesis genetically, we used the retroviral complementation system to ectopically express p100 in both control and Syk-deficient B cells (Figure 3C). We found that increased expression of p100 did not rescue in vivo survival of Syk-deficient B cells, did not affect the increased amounts of surface IgM and did not rescue BAFF-dependent survival in vitro (Figures 3D–3H). In contrast, control B cells overexpressing p100 survived much better in vitro in response to BAFF compared to uninfected cells, demonstrating that the ectopically expressed p100 was biologically functional.

To explore this pathway further, we made use of a mutant form of p100 (p100ΔC), which is converted more efficiently to p52 (Figure S3A) (Liao and Sun, 2003). Once again the retroviral complementation assay showed that ectopic expression of p100ΔC was not able to increase survival of Syk-deficient B cells in vivo, normalize IgM amounts, or rescue the defect in BAFF-induced survival in vivo (Figures S3B–S3E). Thus we conclude that the failure of Syk-deficient B cells to survive is not due to a defect in the p100 pathway.

### Normal Expression of Bcl-2-Family Proteins in Syk-Deficient B Cells

Stimulation of B cells with BAFF leads to the upregulation of several antiapoptotic members of the Bcl-2-family of proteins, including Bcl-2, Bcl-xL, Mcl1, and A1, and to the downregulation of the proapoptotic Bim and Bad proteins (Rickert et al., 2011). To investigate whether dysregulation of these proteins contributed to the survival defect of Syk-deficient B cells, we initially measured mRNA amounts for Bcl-2-family proteins (Table S1). We detected no significant changes in any of these, except for the genes encoding A1, which were expressed at around 60% of the amount in control B cells. To explore this further, we immunoblotted cell lysates from control and mutant B cells and probed for several Bcl-2-family members, including Bcl-2, Bcl-xL, Mcl1, A1, Bim, Bid, Bad, and Bax. We saw no consistent differences between mutant and control B cells in the amounts of any of these proteins, including A1 (Figures S3F and S3G), suggesting that reduced survival of Syk-deficient B cells is not caused by dysregulated expression of Bcl-2-family proteins, though we cannot rule out that there may be alterations in posttranslational modification of these proteins that contribute to increased cell death. Because the expression of many of these proteins is under the control of NF-κB transcription factors (Mackay et al., 2010), these results further support the conclusion that the reduced survival of Syk-deficient B cells is unlikely to be due to defects in NF-κB pathways.

### Ectopic Activation of the ERK Pathway Partially Rescues Survival of Syk-Deficient B Cells

**Figure 4:**
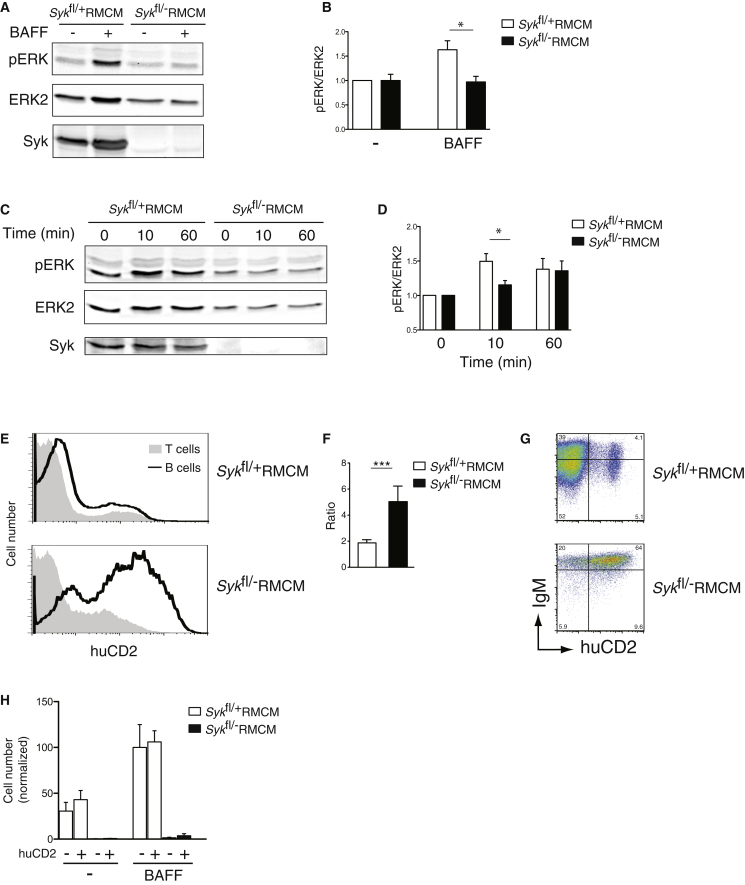
Ectopic Expression of Constitutively Active MEK1 (caMEK1) Rescues In Vivo Survival Defect of Syk-Deficient B Cells (A–D) Splenic B cells of the indicted genotypes were cultured (A and B) for 16 hr in the absence (-) or presence (+) of BAFF or (C and D) for the indicated times. (A and C) Immunoblots of total cell extracts probed with antibodies to pERK, ERK2, and Syk. (B and D) Graphs of mean (±SEM) ratio of pERK to ERK2 in splenic B cells cultured and analyzed as described in (A) and (C), respectively. Ratios were normalized to control B cells cultured without BAFF. (E–H) Radiation chimeras were reconstituted with bone marrow of *Syk*^fl/+^RMCM or *Syk*^fl/−^RMCM mice infected with retrovirus expressing caMEK1 and huCD2, then treated with tamoxifen to induce deletion of Syk. (E) Flow cytometric analysis of huCD2 expression in donor splenic B and T cells from radiation chimeras. (F) Graph of mean (±SEM) ratio of the percentage of donor B cells that were huCD2^+^ to the percentage of donor T cells that were huCD2^+^ in the spleens of chimeric mice reconstituted with bone marrow of the indicated genotypes. (G) Expression of IgM and huCD2 on donor-derived splenic B cells from radiation chimeras. (H) Graph showing mean (±SEM) number of donor B cells from chimeras reconstituted with the indicated genotypes surviving after 4 days culture in the absence (-) or presence of BAFF. Cells were subdivided according to expression of huCD2. Numbers are normalized to the number of surviving control huCD2^−^ B cells in presence of BAFF. See also Figure S4.

Another critical pathway controlling B cell survival is one leading to the activation of the ERK MAP kinases (Craxton et al., 2005). Because Syk transduces BCR signals to the activation of ERK following crosslinking of the BCR, it is possible that it may also transduce the tonic BCR survival signal to ERK activation. Indeed, we found that BAFF-induced phosphorylation of ERK, a hallmark of its activation, was reduced in Syk-deficient B cells (Figures 4A–4D). To investigate whether this defect may contribute to the reduced survival of Syk-deficient B cells, we used the retroviral complementation system to ectopically express a constitutively active MEK1 kinase (caMEK1) a direct activator of the ERK kinases, in both control and mutant B cells. We found that expression of caMEK1 selectively increased survival of Syk-deficient B cells in vivo, while having no effect on control B cells (Figures 4E and 4F). However it did not normalize IgM amounts and did not rescue the defective BAFF-induced survival in vitro (Figures 4G and 4H). The expression of caMEK1 did not lead to aberrant activation of the B cells as shown by unaltered cell size and expression of the activation markers CD69, CD86, and I-A (Figure S4). These results suggest that Syk transduces BAFFR signals to ERK activation and that reduced ERK activation in Syk-deficient B cells may partially account for their decreased survival in vivo.

### Activation of the PI3 Kinase Pathway Partially Rescues Survival of Syk-Deficient B Cells

**Figure 5:**
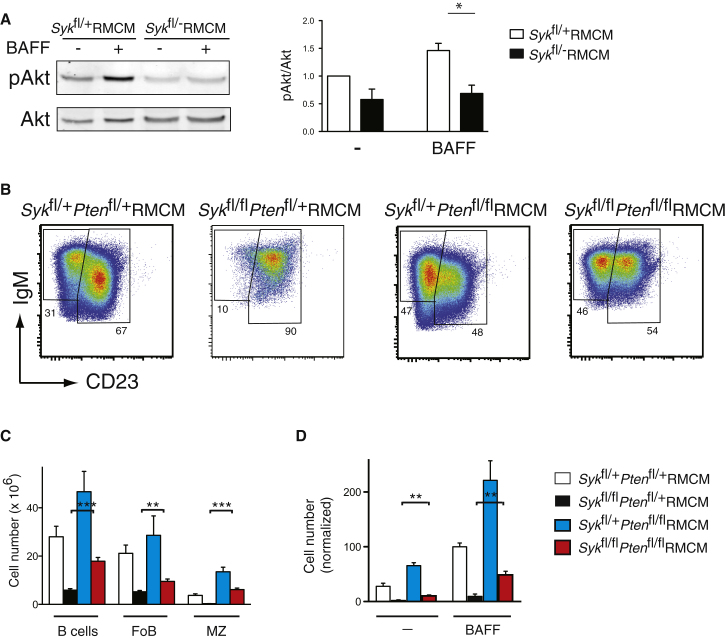
Deletion of *Pten* Rescues In Vivo and In Vitro Survival Defect of Syk-Deficient B Cells (A) Splenic B cells of the indicated genotypes were cultured for 16 hr in the absence (-) or presence (+) of BAFF. Immunoblots of total cell extracts were probed with antibodies to pS473-Akt (pAkt) and Akt. Graph shows mean (±SEM) ratio of pAkt to Akt in splenic B cells cultured with or without BAFF. Ratios were normalized to control B cells cultured without BAFF. (B–D) Radiation chimeras were generated by reconstituting irradiated Rag1-deficient mice with bone marrow from mice carrying conditional alleles of *Syk*, *Pten*, or both; all contained the *Rosa26*^MerCreMer^ allele. Six weeks after reconstitution, chimeras were treated with tamoxifen and analyzed 3 weeks later. (B) Flow cytometric analysis of donor-derived splenic mature B cells (B220^+^CD93^−^) from chimeras reconstituted with marrow of the indicated genotypes. Numbers indicate percentage of B cells in the follicular (FoB, IgM^+^CD23^+^) and marginal zone (IgM^+^CD23^−^) compartments. (C) Mean (±SEM) numbers of all B cells, FoB, and MZ cells in the spleens of the chimeras. (D) Graph showing mean (±SEM) number of B cells of the indicated genotypes surviving after 4 days culture in the absence (-) or presence of BAFF, normalized to the number of surviving control B cells in presence of BAFF. Statistically significant differences are shown only for comparisons between Syk-deficient cells and cells deficient in both Syk and Pten.

A critical pathway controlling survival of many cell types is the PI3 kinase pathway, leading to the production of phosphatidylinositide-3,4,5-trisphosphate (PIP_3_). Previous work has shown that the death of B cells that have lost the BCR can be reversed by ectopic activation of the PI3 kinase pathway, leading to the proposal that the BCR survival signal uses this pathway (Srinivasan et al., 2009). To investigate whether Syk may transduce such a signal, we examined BAFF-induced phosphorylation of Akt as a surrogate for the production of PIP_3_, in both mutant and control B cells. We found that the amount of phosphorylated Akt was reduced in Syk-deficient B cells both before and after stimulation with BAFF (Figure 5A), consistent with Syk transducing both the BCR survival signal and a BAFFR signal leading to activation of PI3 kinase.

To determine whether the reduced activation of PI3 kinase contributes to the survival defect of Syk-deficient B cells, we generated radiation chimeras reconstituted with bone marrow from mice carrying *Rosa26*^MerCreMer^ and a conditional allele of *Syk*, *Pten*, or both. PTEN is a phosphatase that converts PIP_3_ to phosphatidylinositide-4,5-bisphosphate, thereby directly counteracting the activity of PI3 kinases. Deletion of *Pten* will cause a rise in cellular PIP_3_ amount, and thus if loss of Syk-deficient B cells is due in part to reduced PI3 kinase activity, deletion of *Pten* should rescue survival. This is indeed what we observed: loss of PTEN in Syk-deficient B cells resulted in increased numbers of both follicular and marginal zone B cells and partially rescued BAFF-induced survival of Syk-deficient B cells (Figures 5B–5D). Thus we conclude that Syk transduces signals to the PI3 kinase pathway, which are in part required for B cell survival.

### PDK1 Is Essential for B Cell Survival and Response to BAFF

**Figure 6:**
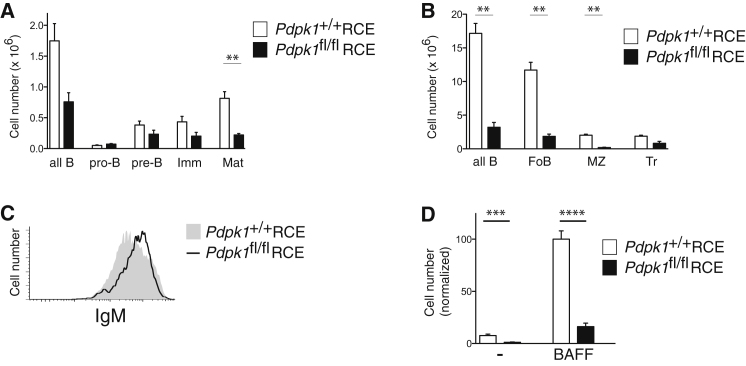
Deletion of PDK1 Leads to the Loss of Most B Lineage Cells Radiation chimeras reconstituted with bone marrow from mice carrying a tamoxifen-inducible Cre (*Rosa26*^Cre-ERT2/+^, RCE) and either wild-type or conditional alleles of the *Pdpk1* gene (*Pdpk1*^+/+^ or *Pdpk1*^fl/fl^), which codes for the PDK1 protein, were treated with tamoxifen and analyzed 6 weeks later. (A) Graph of mean (±SEM) number of all B lineage cells, pro-B, pre-B, immature B (Imm), and mature (Mat) B cells in the bone marrow of indicated chimeric mice. Cell populations are identified as in Figures 1B and 1C. (B) Graph of mean (±SEM) number of all B lineage cells, follicular (FoB), marginal zone (MZ), and transitional (Tr) B cells in the spleen of indicated chimeric mice. Cell populations are identified as in Figures 1D and 1E. (C) Flow cytometric analysis of IgM expression on the surface of splenic B cells from chimeric mice. (D) Graph showing mean (±SEM) number of B cells of the indicated genotypes surviving after 4 days culture in the absence (-) or presence of BAFF, normalized to the number of surviving control B cells in presence of BAFF.

The PI3 kinase pathway contributes to cell survival in part through the activation of Akt and subsequent phosphorylation of the FOXO transcription factors (Zhang et al., 2011). The activation of Akt requires the prior PIP_3_-dependent activation of the PDK1 kinase (encoded by the *Pdpk1* gene) and subsequent phosphorylation of Akt on Thr308 by PDK1 and on Ser473 by mTORC2 or other kinases (Bozulic and Hemmings, 2009). If the requirement for PI3 kinase activation for B cell survival requires Akt activation, then B cell survival may also be dependent on PDK1. To address this possibility, we used bone marrow from mice with a conditional allele of *Pdpk1* (*Pdpk1*^fl^) crossed to a tamoxifen-inducible Cre recombinase expressed from the ROSA26 locus (*Rosa26*^Cre-ERT2/+^, RCE) to reconstitute irradiated mice. Analysis showed that 6 weeks after tamoxifen treatment there was a small reduction in pre-B and immature B cells in the bone marrow, but a large decrease in mature recirculating B cells in the marrow, as well as transitional, follicular, and marginal zone B cells in the spleen (Figures 6A and 6B). Furthermore, the surviving PDK1-deficient B cells had elevated amounts of IgM, and were very defective in their ability to survive in response to BAFF in vitro (Figures 6C and 6D). Thus PDK1 is also essential for the survival of mature B cells and for responses to BAFF, and the phenotype of PDK1-deficient B cells is similar to that of Syk-deficient cells, consistent with the hypothesis that Syk transduces survival signals through PI3 kinase and PDK1.

### BAFFR Signals through the BCR

**Figure 7:**
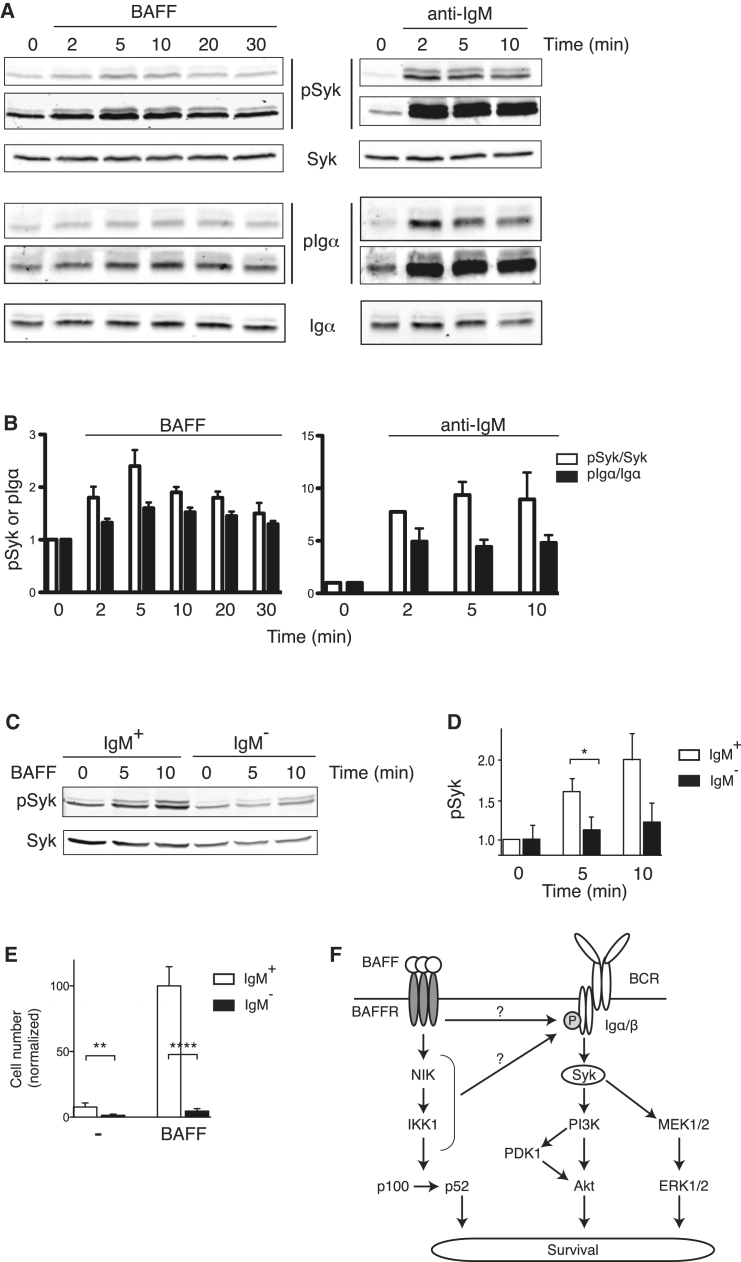
BCR-Dependent BAFF-Induced Phosphorylation of Syk and Igα in B Cells (A) Immunoblots of total cell extracts from splenic B cells stimulated with anti-IgM or BAFF for the indicated times, probed with antibodies to phosphorylated Syk (pSyk), Syk, phosphorylated Igα (pIgα), and Igα. Two different exposures of the same blots are shown for the pSyk and pIgα to show the different magnitudes of the BAFF and anti-IgM responses. (B) Graphs show the mean (±SEM) amounts of pSyk or pIgα in B cells stimulated with anti-IgM or BAFF. The amounts of phosphoproteins were normalized to the amount of Syk or Igα and further normalized to the signal in unstimulated cells at time = 0. (C) Immunoblot of total cell extracts from splenic B cells (either IgM^+^ or IgM^−^) stimulated with BAFF for the indicated times, probed with antibodies to pSyk and Syk. IgM^+^ and IgM^−^ B cells were purified from *Rag1*^−/−^ radiation chimeras reconstituted with bone marrow from *Igh*^B1-8f/+^Tg(Eμ-BclxL) and *Igh*^B1-8f/+^Tg(Eμ-BclxL)RMCM mice respectively, that had been treated with tamoxifen 13–18 days earlier. (D) Graph of mean (±SEM) amounts of pSyk in IgM^+^ and IgM^−^ B cells determined as in (C), normalized to amounts of Syk and to the signal in IgM^+^ B cells at time = 0. (E) Graph showing mean (±SEM) number of IgM^+^ or IgM^−^ B cells surviving after 4 days culture in the absence (-) or presence of BAFF, normalized to the number of surviving IgM^+^ B cells in presence of BAFF. IgM^+^ and IgM^−^ B cells were purified from *Igh*^B1-8f/+^ and *Igh*^B1-8f/+^RMCM mice respectively, that had been treated with tamoxifen 6 days earlier. (F) Proposed signaling pathways from BAFFR and BCR controlling B cell survival, showing BAFFR-induced phosphorylation of Igα and activation of Syk via the BCR. As discussed in the text and indicated by question marks, it is unclear whether BAFFR-induced phosphorylation of Igα occurs via NIK or IKK1 or if the signal is independent of these kinases. See also Figure S5.

The previous results have shown that Syk transduces signals critical for survival of B cells in vivo and BAFF-induced survival in vitro, and it does so in part through ERK and PI3 kinase-PDK1 pathways. However it is unclear from this whether Syk transduces a tonic BCR signal that is required in conjunction with BAFFR signals for cell survival, or whether it might transduce BAFFR signals directly. The latter possibility is suggested by the observation that BAFFR-induced activation of ERK and PI3 kinase pathways is defective in Syk-deficient cells. To investigate this further, we measured BAFF-induced phosphorylation of Syk, a hallmark of its activation. We were able to detect rapid activation of Syk in response to BAFF, peaking at 5 min (Figures 7A and 7B). The increase in Syk phosphorylation in response to BAFF was smaller than that seen following stimulation of the BCR with an anti-IgM antibody (2.5-fold compared to 10-fold); nonetheless, the increase was reproducible and significant.

Syk has been reported to transduce signals from different classes of receptors, including antigen receptors, integrins, and C-type lectins (Mócsai et al., 2010). Despite this variety, in all cases, the activation process involves binding of the SH2 domains of Syk to phosphorylated tyrosines within ITAM-like motifs. In view of this, we postulated that the activation of Syk downstream of BAFFR is also likely to involve a phosphorylated ITAM, with the most obvious candidates in a B cell being the Igα and Igβ proteins. Indeed, we detected phosphorylation of Igα in response to BAFF, peaking around 5 min, similar to the kinetics observed for the phosphorylation of Syk (Figures 7A and 7B). Once again, the increase in Igα phosphorylation was smaller than that seen following BCR stimulation but was nonetheless reproducible and significant.

These results suggested that BAFFR signaling induces Igα phosphorylation, which in turn leads to Syk activation. If this hypothesis is correct, the prediction would be that B cells that had lost the BCR would also lose BAFF-induced Syk phosphorylation. To investigate this, we made use of the B1-8f allele (*Igh*^B1-8f/+^) in which a rearranged VDJ segment flanked by loxP sites is placed in the Ig heavy-chain locus in such a way that the B cells predominantly express this one prerearranged heavy chain (Lam et al., 1997). Cre-induced deletion of the VDJ segment results in the loss of Igμ heavy chain expression and the associated Ig light chains and Igα and Igβ proteins are no longer transported to the cell surface. We generated mice containing both the *Igh*^B1-8f/+^ allele and the tamoxifen-inducible *Rosa26*^MerCreMer^ allele, and treated adult mice with tamoxifen. As expected, this resulted in rapid loss of cell-surface BCR from many, though not all B cells. We purified IgM^−^ B cells from these mice and measured phosphorylation of Syk in response to BAFF compared to IgM^+^ B cells from control mice. Whereas IgM^+^ B cells again showed clear induction of Syk phosphorylation, this was largely absent in IgM^−^ B cells, demonstrating that the BCR was required for BAFF-induced Syk activation (Figures 7C and 7D). Furthermore, we examined the consequence of loss of BCR on BAFF-induced survival. We found that, in contrast to IgM^+^ B cells, IgM^−^ B cells were unable to survive in response to BAFF (Figure 7E). Phosphorylation of Igα and Igβ and subsequent Syk activation following antigen binding to the BCR may be mediated by Src-family kinases (SFKs). Thus we hypothesized that SFKs may also be required for BAFF-induced Syk activation. In agreement with this, treatment of B cells with PP1, a SFK inhibitor, resulted in reduced BAFF-induced Syk phosphorylation (Figures S5A and S5B). Furthermore BAFF-induced B cell survival was inhibited by PP1 and two other SFK inhibitors: PP2 and Src-I (Figure S5C). Taken together, these results show that BAFFR signaling leads to Syk activation via the BCR, potentially through SFK-mediated phosphorylation of Igα, and suggest that this pathway may be required for BAFF-mediated B cell survival (Figure 7F).

## Discussion

Inducible elimination of Syk or of its kinase activity leads to the loss of most follicular and MZ B cells, while sparing the majority of B1 cells. This disappearance of B cells is accompanied by loss of responsiveness to BAFF, which likely accounts for the selective loss of follicular and MZ B cells compared to B1 cells, because a very similar phenotype is seen in mice deficient in BAFF or BAFFR (Mackay et al., 2010). This similarity also supports our proposal that Syk transduces survival signals from BAFFR. It is unlikely that this unresponsiveness to BAFF is simply the result of selective survival of a subset of B cells that cannot respond to BAFF, because the decrease in BAFF-induced survival parallels the loss of Syk and is seen before any substantial decrease in B cell numbers. Nonetheless, we note that a small number of follicular B cells persist in mice deficient in BAFF or BAFFR, particularly following an inducible inactivation of BAFFR (Keren et al., 2011), similar to that seen in the absence of Syk, pointing to the existence of BAFF- and Syk-independent survival pathways for a minority of follicular B cells.

Given the central role for Syk in transducing BCR signals following antigen binding, we had expected that loss of Syk would lead to a phenotype similar to that seen following removal of the BCR from mature B cells. While the two genetic alterations are similar in terms of leading to loss of most follicular and MZ B cells, they are clearly distinct in their effects on B1 cells, which are largely unaffected by loss of Syk, but disappear following deletion of the BCR (Lam et al., 1997). Furthermore, a small number of Syk-deficient B cells persist for many weeks in the animal, whereas B cells without a BCR are short-lived (Kraus et al., 2004). These differences suggest that the BCR survival signal is transduced partly through Syk and partly through other pathways.

Analysis of survival pathways downstream of Syk showed that BAFF-induced activation of ERK and Akt is decreased in the absence of Syk, and genetic rescue experiments showed that the survival of Syk-deficient B cells could be enhanced by ectopic expression of caMEK1 or by deletion of PTEN. Moreover, loss of PDK1 also leads to the disappearance of most follicular and MZ B cells. Taken together, these results suggest that Syk transduces key survival signals through the ERK and PI3 kinase-PDK1 pathways. In contrast, our results do not support an important role for Syk-mediated regulation of the amounts of either BAFFR or NF-κB2 p100 in B cell survival. Furthermore, the largely normal BAFF-induced regulation of the p100 pathway in Syk-deficient B cells demonstrates that this pathway alone is unable to sustain BAFF-dependent B cell survival.

As with Syk-deficient B cells, the death of BCR-deficient B cells could be rescued by deletion of PTEN consistent with the possibility that the BCR survival signal is transduced at least in part through Syk and PI3 kinase (Srinivasan et al., 2009). In contrast, caMEK1 did not rescue survival of cells that had lost the BCR. The reasons for this difference from Syk-deficient B cells are not known, but one possibility is that Syk may also transduce survival signals independently of the BCR, and that these may be transduced via the ERK pathway. Alternatively, the difference could be due to use of different Cre drivers. In our studies we used the *Rosa26*^MerCreMer^ allele, which induces deletion throughout the mature B cell compartment, whereas Kraus et al. used CD21-Cre, which starts to delete within transitional B cells (Kraus et al., 2004). It may be that the survival pathways used in recently matured B cells are different to those used by the bulk of the mature B cells, with a different dependence on the ERK pathway.

We note that both Syk- and BCR-deficient B cells whose survival has been rescued by ectopic activation of the PI3 kinase pathway remain dependent on BAFF for in vitro survival (Figure 5D; Srinivasan et al., 2009). These observations support a model in which BAFFR delivers some critical survival signals independent of the BCR, Syk, and PI3 kinase; these could be through the IKK1-p100 pathway.

Most unexpectedly, our results show that BAFFR signaling leads to phosphorylation of Igα and Syk and that the phosphorylation of Syk is dependent on the BCR, supporting our proposed model that survival signals from BAFFR are transduced via the BCR complex to the activation of Syk. Syk transduces signals downstream of many receptors, including antigen receptors, C-type lectins, and integrins, and in most cases Syk activation requires binding of the kinase to a phosphorylated ITAM (Mócsai et al., 2010). Our data suggest that in the case of BAFFR the activation of Syk depends on the phosphorylation of Igα and possibly Igβ, though we cannot exclude that other ITAM-bearing molecules may be involved. It remains unknown how signaling from BAFFR leads to phosphorylation of Igα, but it may be via SFKs. This is supported by our observation that inhibition of SFKs leads to reduced BAFF-induced phosphorylation of Syk and B cell survival. It is also possible that the signal from BAFFR to the BCR goes through the NIK and/or IKK1 kinases, though we note that NIK activation is induced more slowly by BAFF than the phosphorylation of Syk, making this less likely.

Previous studies had shown that the BCR and its associated Igα subunit were essential for the survival of mature B cells, and these observations led to the suggestion that the BCR may transduce survival signals either following low-affinity interactions of the BCR with self-antigens, or by continuous low-level tonic BCR signaling in the absence of ligand engagement (Kraus et al., 2004; Lam et al., 1997). However, our current results suggest an alternative interpretation. We propose instead that the requirement for the BCR and Igα in B cell survival may be due to a function for these proteins as critical adapters in a BAFFR signaling pathway leading to the activation of Syk and hence to the activation of ERK and PI3 kinase pathways. Based on our data we cannot exclude the possibility that the BCR delivers a stand-alone survival signal; however, we note that in contrast to our proposal, such a model does not explain why BAFFR transduces signals via the BCR to the activation of Syk, ERK, and Akt.

## Experimental Procedures

For an extended description of experimental procedures see Supplemental Information.

### Mice

Gene targeted mice were bred in an SPF facility at NIMR. For induction of Cre expression, mice were treated with tamoxifen (2 mg/day) for 5 days. Radiation chimeras were generated using standard protocols. For retrovirus-mediated gene transfer, bone-marrow cells were infected in vitro with retroviruses prior to reconstitution of irradiated recipients. If required, chimeric mice were treated with tamoxifen 6–8 weeks after reconstitution.

### Immunoblotting

Splenic B cells were purified by depletion with antibodies to CD43 and CD1d, and lysates were prepared using RIPA buffer. Immunoblotting was performed using standard procedures.

### B Cell Survival Assay

Purified splenic B cells were cultured for 4 days with or without BAFF. Absolute numbers of live B cells were assessed using ToPro3 exclusion.

### RNA Analysis

Follicular B cells (B220^+^CD93^−^CD23^+^IgM^+^) were sorted from spleens 10 days after start of tamoxifen treatment. RNA was isolated using RNEasy mini kit (QIAGEN) and used for microarray or RNASeq analysis.

### Statistical Analysis

All statistical comparisons were carried out using the nonparametric two-tailed Mann-Whitney test. Statistically significant differences are indicated on the figures: ^∗^p < 0.05, ^∗∗^p < 0.01, ^∗∗∗^p < 0.001, ^∗∗∗∗^p < 0.0001.

## Supplemental Information

Document S1. Figures S1–S5, Table S1, and Supplemental Experimental Procedures
