# Supplementary material for: The BAFF Receptor Transduces Survival Signals by Co-opting the B Cell Receptor Signaling Pathway
Source: Immunity. 2013 Mar 21;38(3):475–88. doi: 10.1016/j.immuni.2012.11.015 (PMC3627223; doi:10.1016/j.immuni.2012.11.015)
Supplement: Document S1. Figures S1–S5, Table S1, and Supplemental Experimental Procedures [file mmc1.pdf]

Immunity, Volume *38*

Supplemental Information

The BAFF Receptor Transduces Survival Signals by Co-opting the B Cell Receptor Signaling Pathway

Edina Schweighoffer, Lesley Vanes, Josquin Nys, Doreen Cantrell, Scott McCleary, Nicholas Smithers, and Victor L.J. Tybulewicz

Supplemental Inventory

1. Supplemental Figures and Tables

Figure S1, related to Figure 1

Figure S2, related to Figure 2

Figure S3, related to Figure 3

Figure S4, related to Figure 4

Figure S5, related to Figure 7

Table S1, related to Figure 3

2. Supplemental Experimental Procedures

3. Supplemental References

Figure S1

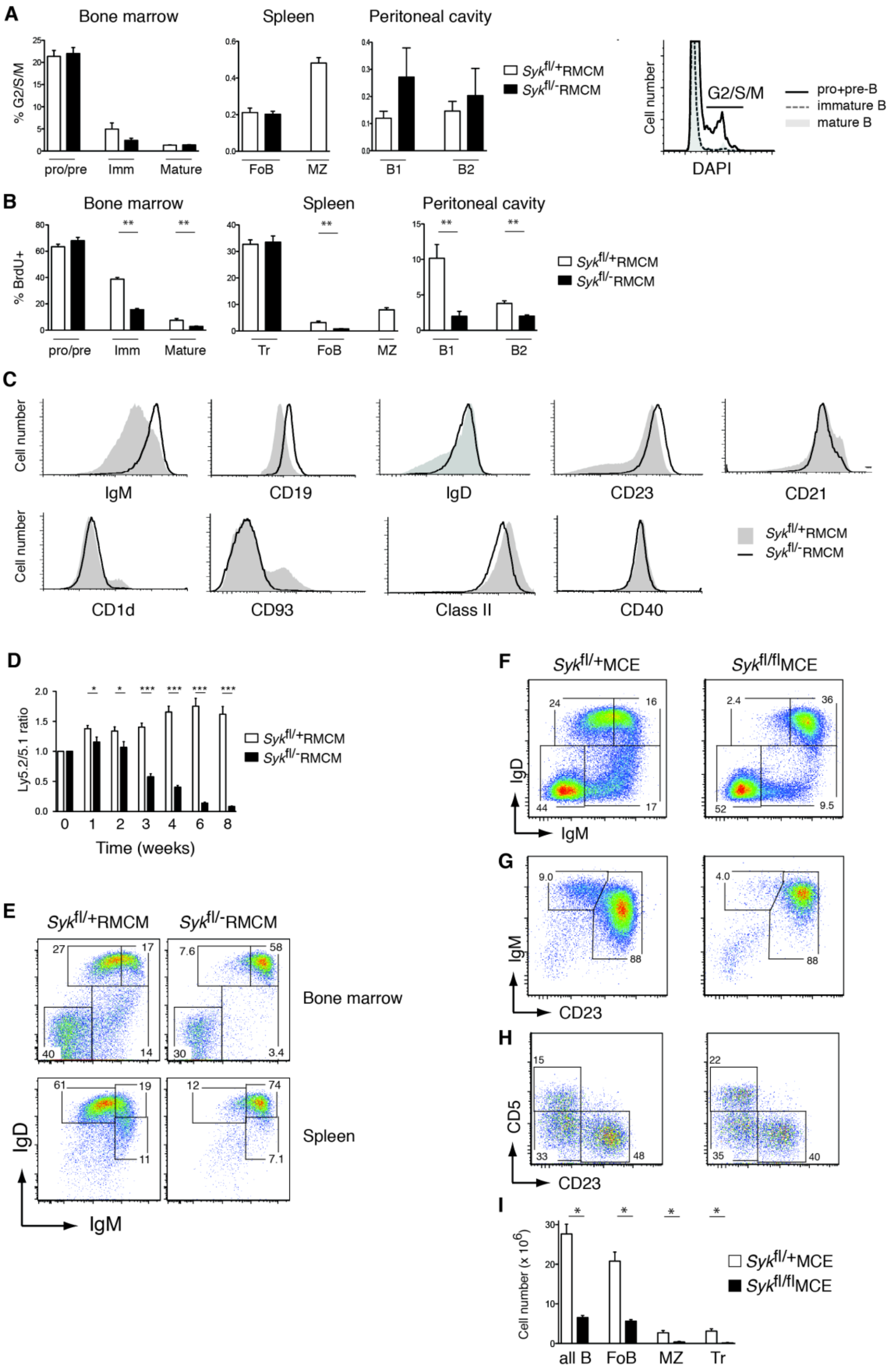

A
Bone marrow
Spleen
Peritoneal cavity
% G2/S/M
pro/pre
Imm
Mature
FoB
MZ
B1
B2
Sykfl/+RMCM
Sykfl/-RMCM
Cell number
G2/S/M
DAPI
pro+pre-B
immature B
mature B
B
Bone marrow
Spleen
Peritoneal cavity
% BrdU+
pro/pre
Imm
Mature
Tr
FoB
MZ
B1
B2
Sykfl/+RMCM
Sykfl/-RMCM
C
Cell number
IgM
CD19
IgD
CD23
CD21
CD1d
CD93
Class II
CD40
Sykfl/+RMCM
Sykfl/-RMCM
D
Ly5.2/5.1 ratio
Time (weeks)
Sykfl/+RMCM
Sykfl/-RMCM
E
Sykfl/+RMCM
Sykfl/-RMCM
Bone marrow
Spleen
IgD
IgM
F
Sykfl/+MCE
Sykfl/flMCE
IgD
IgM
G
IgM
CD23
H
CD5
CD23
I
Cell number (x 10^6)
all B
FoB
MZ
Tr
Sykfl/+MCE
Sykfl/flMCE

Figure S1. Surviving Syk-Deficient B Cells Are Quiescent, Long-Lived and Resemble Mature Follicular B Cells, and the Requirement for Syk Is Cell Autonomous and Intrinsic to the B Cell Lineage, Related to Figure 1

(**A**) Graphs of mean (±SEM) percentage of cells in G2/S/M phases of the cell cycle are shown for B cell subpopulations of the bone marrow, spleen and peritoneal cavity, determined using staining with DAPI for DNA content. Mice of the indicated genotypes were analyzed 3 weeks after start of tamoxifen treatment. In bone marrow pro/pre-B cells are $B220^{+}CD19^{+}IgD^{-}IgM^{-}$, immature (Imm) B cells are $B220^{+}CD19^{+}IgD^{-}IgM^{+}$, mature B cells are $B220^{+}CD19^{+}IgD^{+}IgM^{+}$; in spleen mature follicular B cells (FoB) are $B220^{+}IgM^{+}CD23^{+}CD21^{+}$, marginal zone (MZ) B cells are $B220^{+}IgM^{+}CD23^{-}CD21^{+}$; peritoneal cavity B1 cells are $B220^{+}IgM^{+}CD23^{-}$, B2 cells are $B220^{+}IgM^{+}CD23^{+}$. Histograms on right show DAPI staining on the indicated subsets of bone marrow cells. Marker indicates cells in G2/S/M phases of cell cycle.

(**B**) Graphs of mean (±SEM) percentage of $BrdU^{+}$ cells are shown for B cell subpopulations of the bone marrow, spleen and peritoneal cavity, from mice of the indicated genotypes analyzed 3 weeks after the start of tamoxifen treatment, with continuous administration of BrdU for the final 7d prior to analysis. Subpopulations were defined as described in (A).

(**C**) Histograms showing expression of indicated proteins on the surface of splenic B cells from mice of indicated genotypes 3 weeks after start of tamoxifen treatment.

(**D, E**) B6.SJL (Ly5.1+) mice were irradiated and reconstituted with a 1:1 mix of bone marrow from B6.SJL (Ly5.1+) and either control (*Syk*$^{fl/+}$RMCM, Ly5.2^{+}) or conditional Syk-deficient (*Syk*$^{fl/-}$RMCM, Ly5.2^{+}) mice. (D) Graph of mean (±SEM) ratio of Ly5.2^{+}/Ly5.1^{+} blood B cells as a function of time after start of tamoxifen treatment, and genotype of the Ly5.2^{+} cells. Ratios were normalized to ratio at time=0. (E) Plots show expression of IgM and IgD on Ly5.1^{-} bone marrow cells and splenocytes, 3 weeks after start of tamoxifen treatment. Numbers indicate percentage of cells falling into gates.

(**F-I**) Control (*Syk*$^{fl/+}$;*Cd79a*$^{CreERT2/+}$, *Syk*$^{fl/+}$MCE) or conditional Syk-deficient mice (*Syk*$^{fl/fl}$;*Cd79a*$^{CreERT2/+}$, *Syk*$^{fl/fl}$ MCE) were treated with tamoxifen and analyzed 3 weeks later. (F) Expression of IgM and IgD on $CD19^{+}B220^{+}$ bone marrow cells distinguishes pro-B and pre-B cells ($IgM^{-}IgD^{-}$), immature ($IgM^{+}IgD^{-}$) and mature ($IgM^{+}IgD^{+}$) B cells. Numbers show percentage of cells in each gate. (G) Expression of IgM and CD23 on $B220^{+}CD93^{-}$ splenocytes identifies follicular ($IgM^{+}CD23^{+}$) and marginal zone ($IgM^{+}CD23^{-}$) B cells. Numbers show percentage of cells in each gate. (H) Expression of CD5 and CD23 on $CD19^{+}B220^{+}$ peritoneal cavity cells identifies B1a ($CD5^{+}CD23^{-}$), B1b ($CD5^{-}CD23^{-}$) and B2 ($CD5^{-}CD23^{+}$) cells. (I) Graph of mean (±SEM) numbers of all splenic B cells, follicular (FoB), marginal zone (MZ) and transitional (Tr) B cells identified as in (G). Transitional B cells were $B220^{+}CD93^{+}$.

Figure S2

A

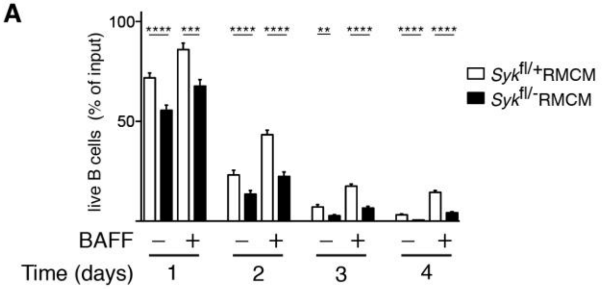

B

Gene Symbol	Gene Description	Corrected p-value	p-value	Fold change (mutant v control)
Hepacam2	HEPACAM family member 2	0.0972	2.60E-05	1.73
Pde2a	phosphodiesterase 2A	0.0175	7.18E-07	1.71
Sep11	septin 11	0.0972	3.26E-05	-1.53
Pcp4	Purkinje cell protein 4	0.0972	4.01E-05	-2.93

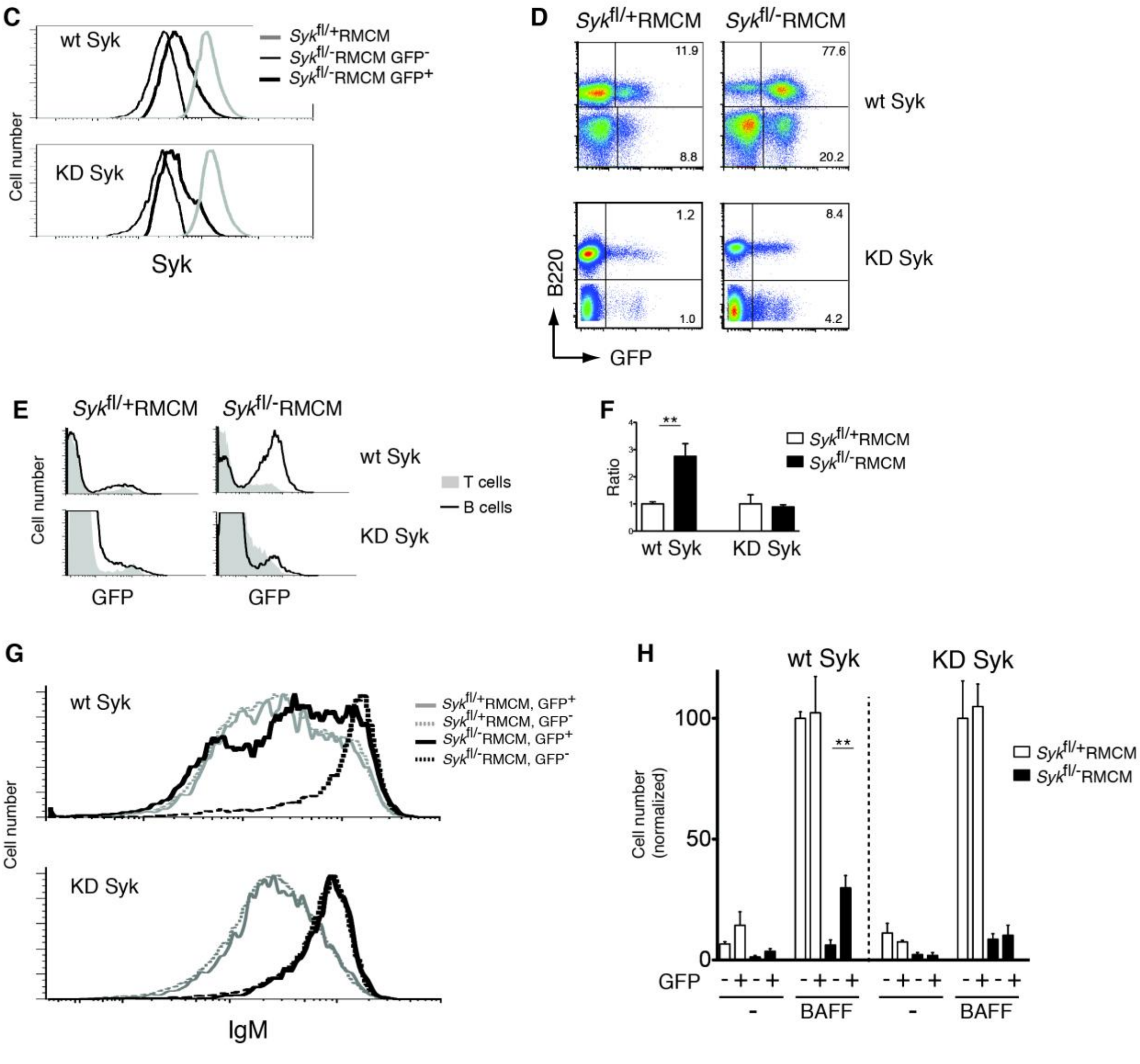

Figure S2. Syk Is Required for *In Vitro* Survival of B Cells in Response to BAFF, and Ectopic Expression of Wild-Type but Not Kinase-Inactive Syk Rescues the *In Vivo* and *In Vitro* Survival Defects of Syk-Deficient B Cells, Related to Figure 2

(**A**) B cells taken from mice of the indicated genotypes treated 10d earlier with tamoxifen, were cultured in the absence or presence of BAFF for 1 – 4d. Graph shows mean (±SEM) number of live B cells remaining in the culture at different time points normalized to the starting input number of B cells, which was set to 100%. Results are from duplicate or triplicate cultures from each of 10 control and 11 mutant mice.

(**B**) Microarray analysis of mRNA expression in splenic B cells from $Syk^{fl/+}$RMCM (control) v $Syk^{fl/-}$RMCM (mutant) mice (n=5 of each) treated 10d earlier with tamoxifen. Table shows all genes whose expression was different between mutant and control B cells with a corrected p-value of <0.1, and a fold change of >1.5. Only 4 out of 21,000 genes passed these thresholds. Table also shows the uncorrected p-values and the fold change of expression between mutant and control B cells; this is a positive value when the mutant was higher and a negative value when expression in the mutant B cells was lower. Note that only one gene (*Pde2a*) was significantly different with a corrected p-value of <0.05.

(**C-H**) Radiation chimeras were reconstituted with bone marrow of $Syk^{fl/+}$RMCM or $Syk^{fl/-}$RMCM mice infected with retrovirus expressing GFP and either wild-type (wt) or kinase dead (KD) Syk, then treated with tamoxifen to induce deletion of Syk, and analyzed 6 weeks later. (C) Flow cytometric analysis of Syk expression in splenic B cells from chimeras reconstituted with Syk-deficient marrow ($Syk^{fl/-}$RMCM) either uninfected (GFP^-) or infected (GFP^+) with retrovirus expressing the indicated Syk protein, compared to uninfected splenic B cells from chimeras reconstituted with control marrow ($Syk^{fl/+}$RMCM). (D) Expression of B220 and GFP on splenic B or T cells from radiation chimeras. Numbers represent percentage of infected cells (GFP^+) within the B ($B220^+$) or T ($B220^-$) cell population. (E) GFP expression in splenic B or T cells from chimeras reconstituted with cells of the indicated genotypes and infected with Syk-expressing retroviruses. Note the selective expansion of Syk-deficient B cells compared to T cells when infected with virus expressing wt Syk. This does not happen with KD Syk or in B cells still expressing endogenous Syk. (F) Graph of mean (±SEM) ratio of the percentage of donor B cells that were GFP^+ to the percentage of donor T cells that were GFP^+ in the spleens of chimeric mice reconstituted with bone marrow of the indicated genotypes. (G) IgM expression on the surface of donor-derived splenic B cells from radiation chimeras of the indicated genotypes infected with Syk-expressing retroviruses. Cells were subdivided according to GFP expression. (H) Graph showing mean (±SEM) number of donor B cells from chimeras reconstituted with the indicated genotypes surviving after 4d culture in the absence (-) or presence of BAFF. Cells were subdivided according to expression of GFP. Numbers are normalized to the number of surviving control GFP^- B cells in presence of BAFF.

Figure S3

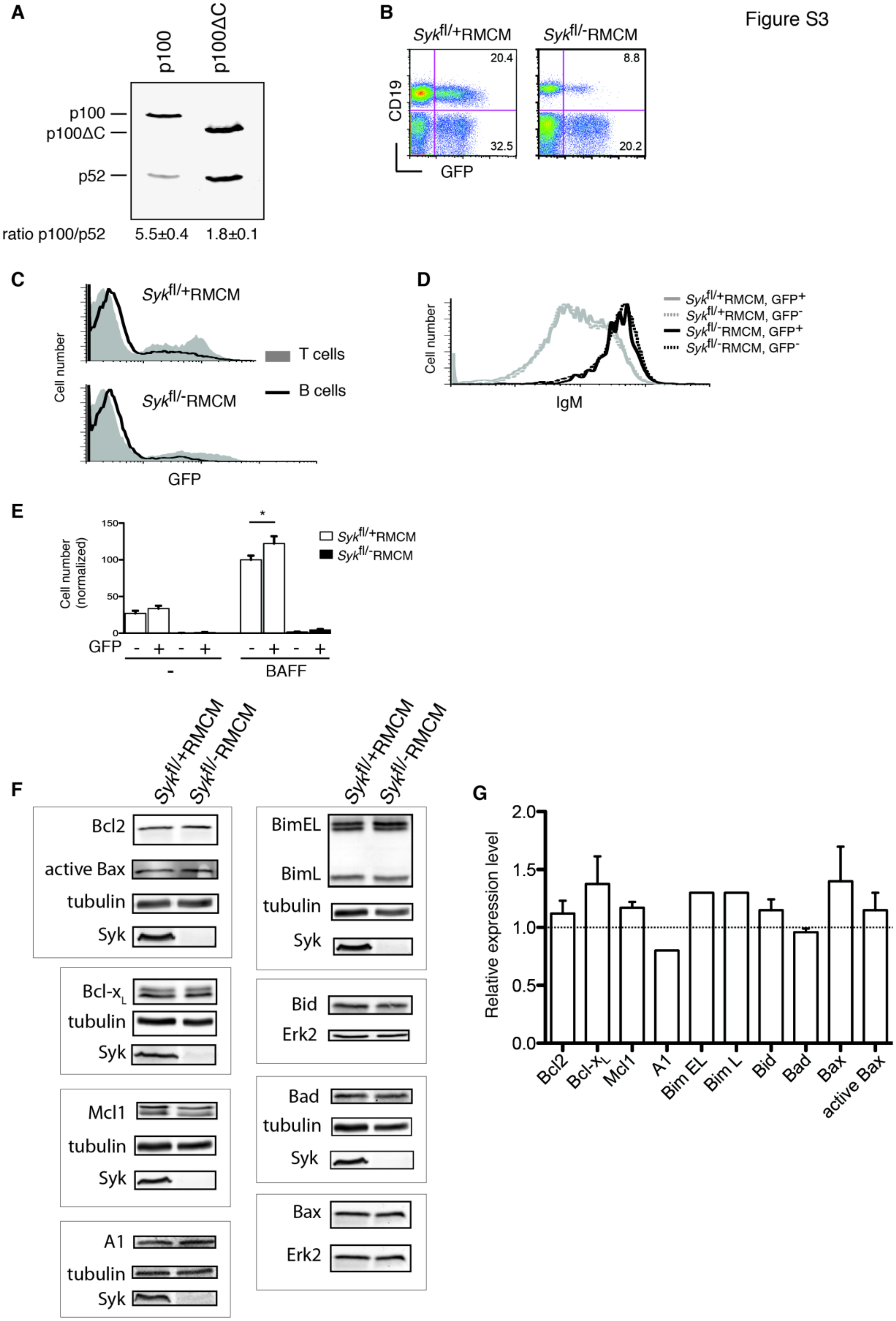
A
p100
p100ΔC
p100
p100ΔC
p52
ratio p100/p52
5.5±0.4
1.8±0.1
B
Sykfl/+RMCM
Sykfl/-RMCM
CD19
GFP
20.4
32.5
8.8
20.2
C
Sykfl/+RMCM
Sykfl/-RMCM
Cell number
GFP
T cells
B cells
D
Cell number
IgM
Sykfl/+RMCM, GFP+
Sykfl/+RMCM, GFP-
Sykfl/-RMCM, GFP+
Sykfl/-RMCM, GFP-
E
Cell number (normalized)
Sykfl/+RMCM
Sykfl/-RMCM
GFP
BAFF
F
Sykfl/+RMCM
Sykfl/-RMCM
Bcl2
active Bax
tubulin
Syk
Bcl-xL
Mcl1
A1
BimEL
BimL
Bid
Erk2
Bad
Bax
G
Relative expression level
Bcl2
Bcl-xL
Mcl1
A1
Bim EL
Bim L
Bid
Bad
Bax
active Bax

Figure S3. Ectopic Expression of p100ΔC Does Not Rescue the *In Vivo* and *in vitro* Survival Defects of Syk-Deficient B Cells, and Unaltered Expression of Bcl-Family Proteins in Syk-Deficient B Cells, Related to Figure 3

(**A**) Immunoblot of total cell extract from 3T3 cells infected with retrovirus expressing either wild-type (p100) or a truncated form of human NFκB2 (p100ΔC, p100(1-753)) and serum-starved for 5 hours. Immunoblot was probed with an antibody to human NFκB2, which recognizes both p100 and p52. Amounts of p100 and p52 were quantitated and the mean (±SEM) p100/p52 ratio from 6 experiments is shown below each lane, demonstrating that p100ΔC is converted more efficiently to p52, than wild-type p100.

(**B-E**) Radiation chimeras were reconstituted with bone marrow of $Syk^{fl/+}$RMCM or $Syk^{fl/-}$RMCM mice infected with retrovirus expressing GFP and p100ΔC, then treated with tamoxifen to induce deletion of Syk, and analyzed 6 weeks later. (B) CD19 and GFP expression in splenic B or T cells from chimeras. Numbers represent percentage of infected cells (as marked by GFP expression) within the B or T cell populations. (C) Histograms of GFP expression on splenic B or T cells in chimeras reconstituted with marrow of the indicated genotypes. (D) IgM expression on the surface of donor-derived splenic B cells from radiation chimeras of the indicated genotypes infected with p100ΔC-expressing retroviruses. Cells were subdivided according to GFP expression. (E) Graph showing mean (±SEM) number of donor B cells from chimeras reconstituted with the indicated genotypes surviving after 4d culture in the absence (-) or presence of BAFF. Cells were subdivided according to expression of GFP. Numbers are normalized to the number of surviving control GFP^- B cells in presence of BAFF.

(**F**) Immunoblots of total cell extracts of splenic B cells from mice of the indicated genotypes that had been treated with tamoxifen 10 days earlier, probed with antibodies against Bcl-family members, against α-tubulin or Erk2 as loading controls and against Syk.

(**G**) Graph showing mean (±SEM) amounts of Bcl-family proteins in B cells from $Syk^{fl/-}$RMCM mice compared to B cells from control $Syk^{fl/+}$RMCM mice. Amounts of proteins were determined from immunoblots such as those in (A), normalized to loading controls and then normalized to amounts in control B cells, which was set to 1. Analysis is from n=2 to 6 for each genotype. No significant differences were found.

Figure S4

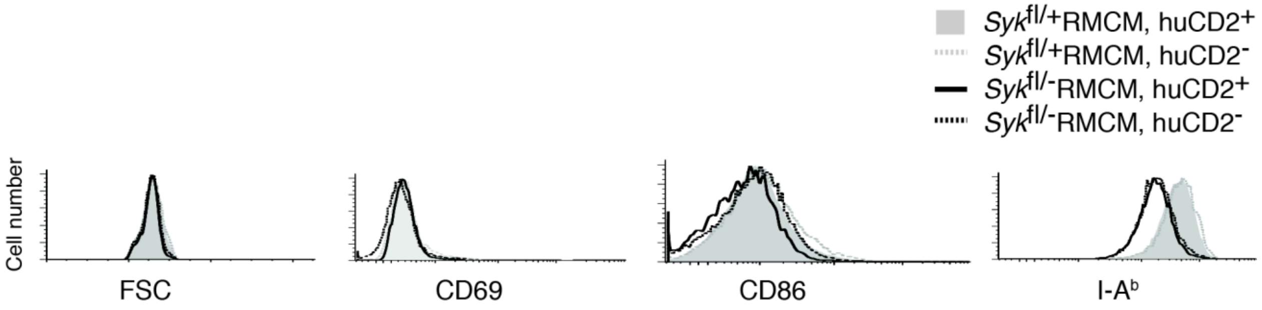

Figure S4. Ectopic Expression of Constitutively Active MEK1 (caMEK1) Does Not Result in Generalized Activation of Syk-Deficient B Cells, Related to Figure 4

Flow cytometric analysis of cell size (forward scatter, FSC) and expression of CD69, CD86 and MHC class II (I-A^{b}) on donor-derived splenic B cells from radiation chimeras reconstituted with bone marrow of *Syk*$^{fl/+}$RMCM or *Syk*$^{fl/-}$RMCM mice infected with retrovirus expressing caMEK1 and huCD2, then treated with tamoxifen to induce deletion of Syk. Cells were subdivided according to expression of huCD2, and hence caMEK1.

Figure S5

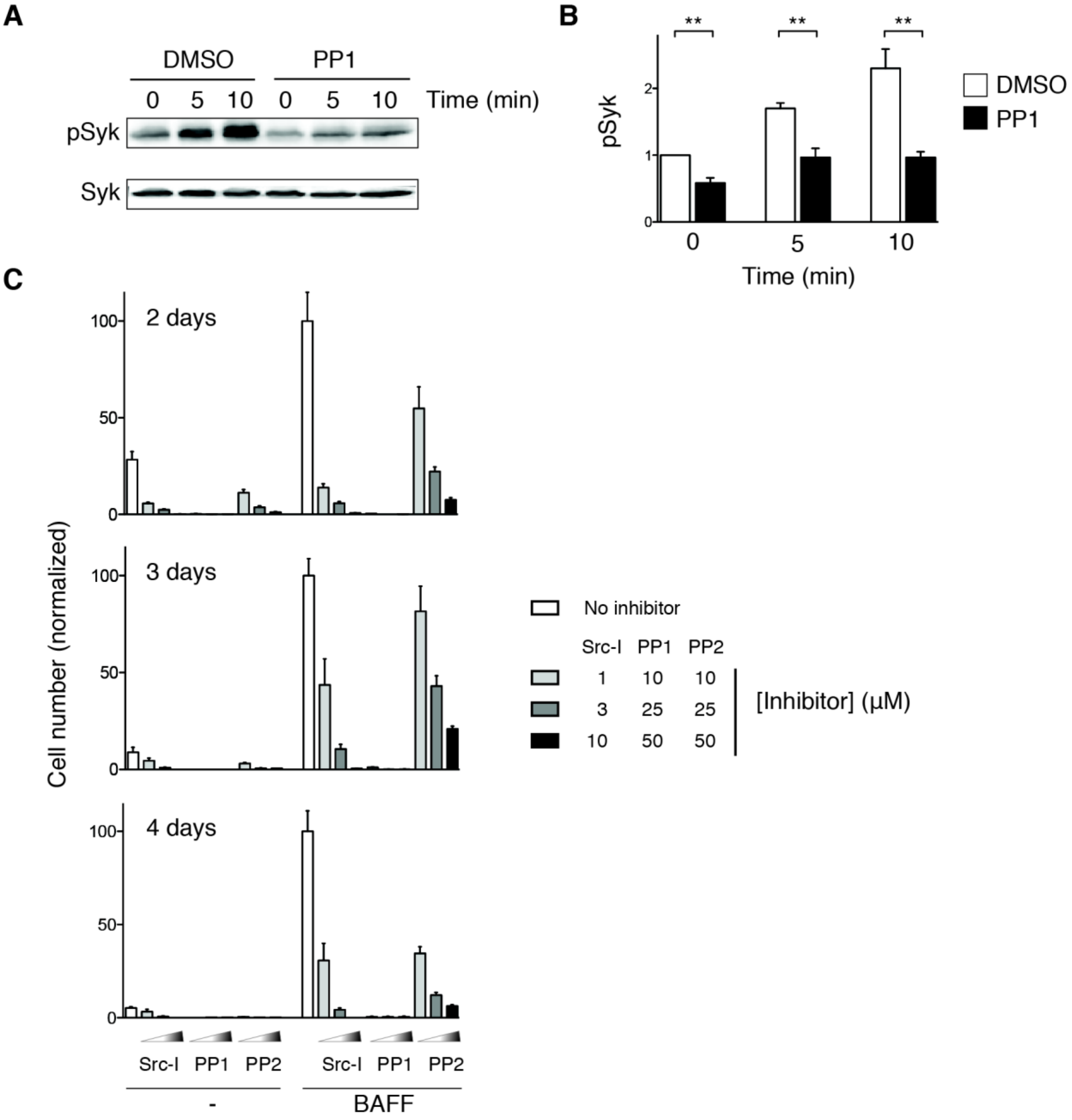
A
DMSO
PP1
0 5 10 0 5 10 Time (min)
pSyk
Syk
B
**
pSyk
DMSO
PP1
0
5
10
Time (min)
C
2 days
3 days
4 days
Cell number (normalized)
No inhibitor
Src-I PP1 PP2
1 10 10
3 25 25
10 50 50
[Inhibitor] (µM)
Src-I PP1 PP2
Src-I PP1 PP2
-
BAFF

Figure S5. Inhibition of Src-Family Kinases Decreases BAFF-Induced Syk Phosphorylation and B Cell Survival, Related to Figure 7

(**A**) Immunoblot of total cell extracts from splenic B cells stimulated with BAFF for the indicated times, in the presence of PP1 (50μM) or vehicle (DMSO) probed with antibodies to pSyk and Syk.

(**B**) Graph of mean (±SEM) amounts of pSyk in B cells stimulated with BAFF for the indicated times in the presence of PP1 or vehicle (DMSO) as in (A), normalized to amounts of Syk and to the signal in B cells treated with vehicle only at time=0.

(**C**) Graph showing mean (±SEM) number of B cells surviving after 2, 3 or 4d culture in the absence (-) or presence of BAFF, and in the absence of inhibitors, or in the presence of the indicated concentrations of Src-family kinase inhibitors Src-I, PP1 and PP2. Cultures without inhibitors received an appropriate amount of vehicle (DMSO). Survival was normalized to the number of surviving B cells in presence of BAFF but no inhibitors.

Gene	Gene symbol	*Syk*$^{fl/+}$RMCM **RPKM**±SEM	*Syk*$^{fl/-}$RMCM **RPKM**±SEM
Mcl-1	Mcl1	**234.55**±2.5	**246.55**±13.0
Bax	Bax	**53.55**±2.6	**47.37**±5.7
Bcl2	Bcl2	**23.31**±1.1	**26.34**±2.5
Bak	Bak1	**20.43**±2.4	**18.26**±1.1
Bfl-1/A1	Bcl2a1b	**19.04**±1.1	**13.16**±0.8
Bid	Bid	**17.09**±1.1	**15.53**±1.6
Bpr	Bcl2l12	**13.94**±1.0	**10.86**±1.2
Bfl-1/A1	Bcl2a1d	**10.95**±1.1	**6.6**±0.12
Bim	Bcl2l11	**10.26**±0.3	**11.40**±0.3
Noxa	Pmaip1	**9.46**±1.8	**7.72**±2.3
Bfl-1/A1	Bcl2a1a	**7.03**±0.04	**4.36**±1.0
Bad	Bad	**6.37**±0.6	**6.38**±0.2
Bcl-w	Bcl2l2	**3.33**±0.01	**3.60**±0.04
Bcl-xl, Bcl-xs	Bcl2l1	**2.52**±0.2	**3.07**±0.2
Puma	Bbc3	**2.15**±0.3	**2.34**±0.2
Bfl-1/A1	Bcl2a1c	**1.35**±0.1	**0.83**±0.2
Bcl-G	Bcl2l14	**0.13**±0.01	**0.22**±0.0
Bik	Bik	**0.04**±0.01	**0.09**±0.03
Mtd	Bok	**0.01**±0.01	**0.02**±0.0
Hrk	Hrk	**0.01**±0.01	**0.0**±0.0

Table S1. Unaltered Expression of Bcl-Family Proteins in Syk-Deficient B Cells, Related to Figure 3

mRNA amounts for Bcl-family proteins in B cells from *Syk*$^{fl/-}$RMCM mice compared to B cells from control *Syk*$^{fl/+}$RMCM mice determined by RNAseq. Results are presented as mean (±SEM) RPKM (reads per KB per million reads).

Supplemental Experimental Procedures

Mice

Mice carrying a conditional allele of *Syk* (*Syk*$^{tm1.1Nns}$, *Syk*^{fl}) with loxP sites in introns 10 and 11, flanking exon 11, and mice with an insertion of a cDNA encoding the MerCreMer fusion protein (Zhang et al., 1996) into the ROSA26 locus, (Gt(ROSA)26Sor$^{tm1(cre/Esr1*)Nns}$, *Rosa26*^{MerCreMer}, RMCM) will be described elsewhere (S. McCleary and N. Smithers, manuscript in preparation). Mice containing a cDNA for CreER^{T2} (Feil et al., 1997) inserted into the *Cd79a* locus that encodes Igα (*Cd79a*$^{tm3(cre/ESR1)Reth}$, *Cd79a*^{CreERT2}, MCE) were a kind gift from M. Reth, University of Freiburg (E. Hobeika and M. Reth, personal communication). Other mouse alleles used: *Pdpk1*$^{tm1.1Mlw}$ (*Pdpk1*^{fl}) (Lawlor et al., 2002), Gt(ROSA)26Sor$^{tm9(cre/ESR1)Arte}$ (*Rosa26*$^{Cre-ERT2/+}$, RCE) (Seibler et al., 2003), *Pten*^{tm1Mro} (Pten^{fl}) (Marino et al., 2002), *Igh*^{tm4Cgn} (IgH^{B1-8f}) (Lam et al., 1997), Tg(Emu-Bcl2l1)#Twb (Tg(Eμ-BclxL)) (Fang et al., 1996), C57BL/6JNimr-*Rag1*^{tm1Mom} (Mombaerts et al., 1992). B6.SJL mice were obtained from the breeding facility at NIMR. This study was conducted following authorization by the UK Home Office, under relevant Project Licence authority.

Tamoxifen and BrdU Treatment of Mice

Mice were injected intraperitoneally for 5 days with 2 mg/day of tamoxifen (Sigma) resuspended at 20 mg/ml in corn oil (Sigma). For some studies, 14 days after start of tamoxifen treatment, *Syk*$^{fl/+}$RMCM and *Syk*$^{fl/-}$RMCM mice were injected intraperitoneally with 1 mg BrdU (Sigma), and then kept on drinking water with 1 mg/ml BrdU for 7 days.

Bone Marrow Chimeras

Bone marrow was harvested from femora and tibiae of donor mice, resuspended in ACK lysis buffer (155 mM NH_4Cl, 10 mM $KHCO_3$, 100 μM EDTA) for 2 min at room temperature to lyse red blood cells, and then injected into recipient mice (at least $1x10^6$ cells/recipient). Recipient Rag1-deficient animals were irradiated with 5Gy using a ^{137}Cs-source, prior to injection. To make mixed chimeras, bone marrow was harvested from *Syk*$^{fl/+}$RMCM or *Syk*$^{fl/-}$RMCM mice, treated with ACK lysis buffer and mixed in a 1:1 ratio with B6.SJL bone marrow cells, and injected at $1x10^6$ cells/recipient into B6.SJL mice that had been irradiated with 2 doses of 4.75Gy from a ^{137}Cs-source. All chimeric animals received Baytril in their drinking water (0.02%, Bayer Healthcare) for at least 4 weeks post-transplantation. If required, 6-8 weeks after reconstitution chimeric mice were treated with tamoxifen.

Retroviral Vectors

pMIGR1 (Pear et al., 1998) and pMIGR1_hu-p100 plasmids were a kind gift from M. Cancro; pMSCV_huCD2 (Clohessy et al., 2004) vector was a gift from Owen Williams (UCL). pMIGR1_hu-p100ΔC was generated by truncating hu-p100 at amino acid 753. pMIGR1_Syk was constructed by inserting an EcoRI-MfeI fragment containing the entire mouse Syk coding sequence including 54 and 66 bases of 5' and 3'UTR, respectively, into the EcoRI site of pMIGR1. Site directed mutagenesis (Quickchange II-E, Stratagene) was used to generate the kinase-inactive Syk mutant (K396R).

pMSCV_BAFFR_huCD2 plasmid was generated by inserting a 971 bp EcoRI fragment from IMAGE clone 40044559 into the EcoRI cloning site of pMSCV_huCD2. pMSCV_caMEK1_huCD2 carries the constitutively active form of human MEK1 (E218, E222) (Cowley et al., 1994) and was a kind gift from Chris Marshall.

Generation of Retroviruses

Transfection mixture of 15µg retroviral vector DNA, 45 µl Genejuice (Merck 70967) and 800µl FCS-free DMEM medium was incubated at room temperature for 45 min before being added dropwise to Plat-E packaging cells (Morita et al., 2000) ($2x10^6$ in a 10cm plate). Supernatant containing retrovirus was harvested 48, 72 and 96 hours after transfection, and concentrated by centrifugation at 15,000*g*. Titers of supernatants were determined by infecting NIH-3T3 cells.

Retroviral Chimeras

Syk$^{fl/+}$RMCM or *Syk*$^{fl/-}$RMCM mice were injected intraperitoneally with 100 mg/kg 5-fluorouracil (Invivogen). Five days later bone marrow was harvested, treated with ACK lysis buffer, then cultured overnight in DMEM-plus medium (DMEM with 10% FCS (Lonza), 100U/ml Penicillin, 100µg/ml Streptomycin, 100µM non-essential amino acids, 20mM HEPES buffer and 100µM 2-mercaptoethanol) containing 100ng/ml rmSCF (Peprotech), 6 ng/ml IL-3 (Sigma) and 10 ng/ml rmIL-6 (Peprotech). 24 hours later cells were moved onto Retronectin-coated plates (Takara Clontech T100B), viral supernatant was added and cultured overnight. This infection step was repeated two more times on consecutive days before cells were harvested and injected intravenously (at least $2.5x10^5$ cells/recipient) into B6.SJL recipient mice that had received 2 doses of 4.75Gy irradiation 3 hours apart. Six weeks after cell transplantation the efficiency of infection was assessed by flow cytometry of peripheral blood, and tamoxifen treatment was started.

Flow Cytometry

Single cell suspensions of bone marrow, spleen, blood or peritoneal wash were treated with ACK lysis buffer to remove red blood cells before staining in FACS buffer (PBS, 0.5% BSA, 0.01%NaN_3, pH 7.2-7.4) containing the appropriate, pre-titered antibodies. Antibodies used, indicating antigen and fluorophore (and clone): CD1d-PE and CD1d-bio (clone 1B1), CD21-FITC (eBio8D9), CD23-PE (B3B4), B220-eFluor450 (RA3-6B2), CD93-APC (AA4.1), IgM-PECy7 (II/41), IgD-FITC and IgD-bio (11-26), Ly5.1-APC (A20), Ly-5.2-FITC (104), bio-CD43 (eBioR2/60), BAFFR-APC (eBio-7H22-E16) were from eBiosciences, CD4-PerCP (RM4-5) and CD8-PerCP (53-6.7) from BioLegend, CD2-PE (RM2-5), CD9-bio (KMC8), CD21-PE (7G6), CD69-FITC (H1.2F3), CD86-PE (GL-1), huCD2-PE (RPA-2.10), Sreptavidin-PerCP (554064) from Becton Dickinson, CD19-APC (RM705) and CD23-APC (MCD2305) from Life Technologies, goat-anti-mouse IgM Fab-FITC (115-097-020) from Jackson Immunoresearch, goat-anti-mouse IgM-FITC (STAR86F) from Serotec and biotinylated goat-anti-mouse-IgM from Southern Biotech.

Intracellular antigens were detected by fixing the cells for 30 min at room temperature with 3% PFA, followed by treatment for 10 min with 50 mM NH_4Cl and permeabilization with 1% NP-40 (Sigma) before adding antibodies or 1µg/ml DAPI (Sigma). BrdU incorporation was detected by intracellular staining with BD Fastimmune anti-BrdU-FITC with DNAse (Becton Dickinson #340649).

B Cell Survival Assay

Splenic B cells were purified by Dynabead depletion, using biotinylated anti-CD43 and anti-CD1d antibodies and Streptavidin-coupled Dynabeads (Life Technologies). Purity of the resulting B cell preparation was assessed by flow cytometry. B cells (10^6/ml) were cultured in DMEM-plus medium with or without 200 ng/ml BAFF (Peprotech). In some cases the SFK inhibitors PP1, PP2 or Src-I were added. Cells were harvested 1 – 4 days later, stained with antibodies to a B cell marker (B220 or CD19), anti-IgM and anti-huCD2 (if required), resuspended in FACS buffer containing PerCP-beads (Becton Dickinson, #340497) and, just before analysis, TO-PRO-3 (Life Technologies) was added to mark dead or dying cells. Number of live B cells per 10,000 beads was determined, and then corrected using the purity data from the input samples. Values were normalized either to *Syk*$^{fl/+}$RMCM B cells cultured in the presence of BAFF or to the number of input B cells.

Immunoblotting

To purify B cells, splenocyte suspensions were treated with ACK lysis buffer, incubated with biotinylated anti-CD43 and anti-CD1d, and then with Streptavidin Dynabeads and labeled cells were removed with a magnet. For the preparation of BCR-deficient B cells, biotinylated anti-IgM and anti-IgD antibodies were also included in the depletion mix. Depleted cells were washed, resuspended in fresh medium, and after at least 5 min at 37°C, BAFF (200 ng/ml) or anti-IgM F(ab')2 (10 µg/ml, Jackson ImmunoResearch) was added for the time indicated. In some cases cells were pre-incubated with PP1 (50µM) for 30 min. Cell lysates were prepared using RIPA buffer. After removal of cell debris by centrifugation, proteins of cell lysates were separated on 10% or 12.5% SDS-PAGE gels and transferred onto Immobilon-FL PVDF membrane (Millipore) by standard techniques. Membranes were blocked for 1 hour in Odyssey Blocking Buffer (Li-Cor #927-40000), and then probed with the following antibodies: anti-phospho-Igα (Y182) rabbit polyclonal, Cell Signaling #5173S; anti-phospho-Syk/ZAP (Y352/Y319) rabbit polyclonal, Cell Signaling #2701L; anti-phospho-Erk1/2 mouse monoclonal, Santa Cruz sc-7383; anti-Erk2 rabbit polyclonal, Santa Cruz sc-154; anti-Syk mouse monoclonal (clone 5F5), Biolegend 646002; anti-NFκB2 (p100/p52) rabbit polyclonal, Cell Signaling #4882; anti-Igα mouse monoclonal (clone 24C2.5), eBioscience #14-0791-82; anti-phospho-Akt (S473) rabbit polyclonal, Cell Signaling, #9271L; anti-Akt mouse monoclonal (B1), Santa Cruz sc-5298; anti-Bcl-2 rabbit monoclonal, clone 50E3, Cell Signaling #28705; anti-Bcl-xL rabbit polyclonal, Santa Cruz sc-634; anti-Bad rabbit polyclonal, Cell Signaling #9292; anti-Bim rabbit polyclonal, Assay Designs #AAP-330; anti-Mcl1 rabbit polyclonal, Rockland #600-401-394; anti-Bok rabbit polyclonal, Cell Signaling #4521; anti-Puma rabbit polyclonal, Cell Signaling #4976; anti-Bax rabbit polyclonal, Cell Signaling #2772; anti-active Bax mouse monoclonal (clone 6A7), Santa

Cruz sc-23959; anti-tubulin mouse monoclonal (clone TAT-1), prepared in-house. Secondary antibodies: Alexa-Fluor 680 goat anti-rabbit IgG (H+L), Invitrogen #A21109; goat anti-mouse IRDye800CW, Licor Biosciences #926-32210. Signals were detected with an Odyssey Infrared Imager (Li-Cor Biotechnology) and analyzed with the manufacturer's software.

RNA Sequencing (RNAseq) and Microarray Analysis of Transcription

Follicular B cells were sorted from the spleens of control (*Syk*$^{fl/+}$RMCM) and conditional Syk-deficient (*Syk*$^{fl/-}$RMCM) mice 10 days after start of tamoxifen treatment using a MoFlo cell sorter (DAKO Cytomation). B220^{+}CD93^{-}CD23^{+}IgM^{+} cells were sorted from control samples, whereas B220^{+}CD93^{-}CD23^{+}IgM^{high} cells were isolated from Syk-deficient spleens to ensure that all sorted cells have deleted the conditional *Syk* allele. RNA was isolated using RNEasy mini kit (Qiagen). Two control and two Syk-deficient libraries were made from 1 µg total RNA each using an mRNA-Seq 8-Sample Prep Kit (Illumina). Samples were analyzed with an Illumina Genome analyzer II, collecting 40 million reads of 35 bases per sample. CLC Genomics Workbench (CLC bio) was used to map the reads to a reference sequence (NCBI37/mm9) and to generate expression values (RPKM).

For microarray analysis, splenic B cells were sorted as described above (n=5 of each genotype), and RNA was isolated using Trizol, then further purified using the RNEasy kit. RNA was labeled, hybridized to Affymetrix Mouse Gene 1.0 ST chips and scanned by UCL Genomics. Data were analyzed using Genespring (Agilent Technologies) for significant changes in gene expression between mutant and control samples using the t test, and resulting p-values were corrected for multiple testing using the Benjamini-Hochberg method.

Statistical Analysis

All statistical comparisons were carried out using the nonparametric two-tailed Mann-Whitney test. Statistically significant differences are indicated on the Figures: *$p < 0.05$, **$p < 0.01$, ***$p < 0.001$, ****$p < 0.0001$.

Supplemental References

Clohessy, J.G., Zhuang, J., and Brady, H.J. (2004). Characterisation of Mcl-1 cleavage during apoptosis of haematopoietic cells. Brit. J. Haematol. *125*, 655-665.

Cowley, S., Paterson, H., Kemp, P., and Marshall, C.J. (1994). Activation of MAP kinase kinase is necessary and sufficient for PC12 differentiation and for transformation of NIH 3T3 cells. Cell *77*, 841-852.

Fang, W., Mueller, D.L., Pennell, C.A., Rivard, J.J., Li, Y.S., Hardy, R.R., Schlissel, M.S., and Behrens, T.W. (1996). Frequent aberrant immunoglobulin gene rearrangements in pro-B cells revealed by a bcl-xL transgene. Immunity *4*, 291-299.

Feil, R., Wagner, J., Metzger, D., and Chambon, P. (1997). Regulation of Cre recombinase activity by mutated estrogen receptor ligand-binding domains. Biochem Biophys Res Commun *237*, 752-757.

Lawlor, M.A., Mora, A., Ashby, P.R., Williams, M.R., Murray-Tait, V., Malone, L., Prescott, A.R., Lucocq, J.M., and Alessi, D.R. (2002). Essential role of PDK1 in regulating cell size and development in mice. EMBO J. *21*, 3728-3738.

Marino, S., Krimpenfort, P., Leung, C., van der Korput, H.A., Trapman, J., Camenisch, I., Berns, A., and Brandner, S. (2002). PTEN is essential for cell migration but not for fate determination and tumourigenesis in the cerebellum. Development *129*, 3513-3522.

Mombaerts, P., Iacomini, J., Johnson, R.S., Herrup, K., Tonegawa, S., and Papaioannou, V.E. (1992). RAG-1-deficient mice have no mature B and T lymphocytes. Cell *68*, 869-877.

Morita, S., Kojima, T., and Kitamura, T. (2000). Plat-E: an efficient and stable system for transient packaging of retroviruses. Gene Ther *7*, 1063-1066.

Pear, W.S., Miller, J.P., Xu, L., Pui, J.C., Soffer, B., Quackenbush, R.C., Pendergast, A.M., Bronson, R., Aster, J.C., Scott, M.L.*, et al.* (1998). Efficient and rapid induction of a chronic myelogenous leukemia-like myeloproliferative disease in mice receiving P210 bcr/abl-transduced bone marrow. Blood *92*, 3780-3792.

Seibler, J., Zevnik, B., Kuter-Luks, B., Andreas, S., Kern, H., Hennek, T., Rode, A., Heimann, C., Faust, N., Kauselmann, G.*, et al.* (2003). Rapid generation of inducible mouse mutants. Nucleic Acids Res. *31*, e12.
